# Highly swelling pH-responsive microgels for dual mode near infra-red fluorescence reporting and imaging†

**DOI:** 10.1039/d0na00581a

**Published:** 2020-08-14

**Authors:** Mingning Zhu, Dongdong Lu, Qing Lian, Shanglin Wu, Wenkai Wang, L. Andrew Lyon, Weiguang Wang, Paulo Bártolo, Mark Dickinson, Brian R. Saunders

**Affiliations:** Department of Materials, University of Manchester, MSS Tower Manchester M13 9PL UK mingning.zhu@manchester.ac.uk dongdong.lu@manchester.ac.uk brian.saunders@manchester.ac.uk; Schmid College of Science and Technology, Chapman University Orange CA 92866 USA; Fowler School of Engineering, Chapman University Orange CA 92866 USA; Department of Mechanical, Aerospace and Civil Engineering, School of Engineering, Faculty of Science and Engineering, University of Manchester Manchester M13 9PL UK; Photon Science Institute, University of Manchester Oxford Road Manchester M13 9PL UK

## Abstract

Near infra-red (NIR) fluorescence is a desirable property for probe particles because such deeply penetrating light enables remote reporting of the local environment in complex surroundings and imaging. Here, two NIR non-radiative energy transfer (NRET) fluorophores (Cy5 and Cy5.5) are coupled to preformed pH-responsive poly(ethylacrylate-methacrylic acid-divinylbenzene) microgel particles (PEA-MAA-5/5.5 MGs) to obtain new NIR fluorescent probes that are cytocompatible and swell strongly. NIR ratiometric photoluminescence (PL) intensity analysis enables reporting of pH-triggered PEA-MAA-5/5.5 MG particle swelling ratios over a very wide range (from 1–90). The dispersions have greatly improved colloidal stability compared to a reference temperature-responsive NIR MG based on poly(*N*-isopropylacrylamide) (PNP-5/5.5). We also show that the wavelength of maximum PL intensity (*λ*_max_) is a second PL parameter that enables remote reporting of swelling for both PEA-MAA-5/5.5 and PNP-5/5.5 MGs. After internalization the PEA-MAA-5/5.5 MGs are successfully imaged in stem cells using NIR light. They are also imaged after subcutaneous injection into model tissue using NIR light. The new NIR PEA-MAA-5/5.5 MGs have excellent potential for reporting their swelling states (and any changes) within physiological settings as well as very high ionic strength environments (*e.g.*, waste water).

## Abstract 2

Near infra-red fluorescent pH-responsive microgels provide two methods for remotely reporting swelling changes using photoluminescence spectroscopy and enable imaging in both cells and tissue.

## Graphical abstract

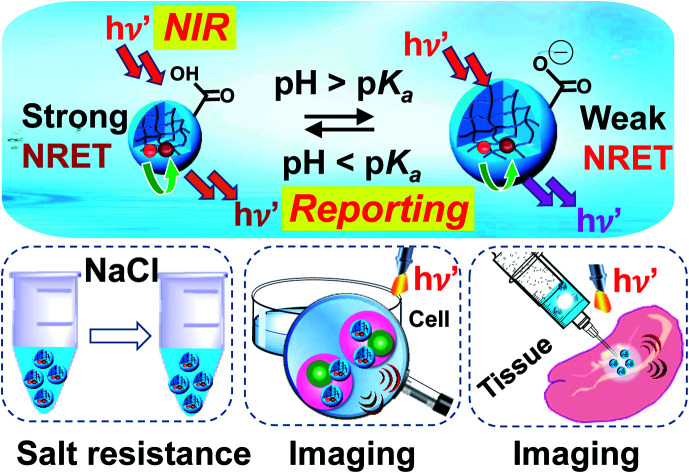

## Introduction

Fluorescent reporting and imaging are invaluable tools for remotely studying complex environments such as those within gels and the body.^1–7^ Fluorescence-based technologies provide relatively simple and inexpensive operating procedures whilst delivering real-time imaging and diagnostic data^8,9^ with high sensitivity,^10,11^ low background noise^12^ and potentially low-cost imaging reagents.^13,14^ The reporter probes studied to date include organic dyes,^15^ fluorescent dots,^16^ plasmonic nanomaterials,^17^ fluorescent proteins^18^ and upconverting nanoparticles.^16^ However, fluorescent probes can have limited sensitivity,^19^ poor spatial resolution,^20^ inadequate stability,^21^ poor biocompatibility or even toxicity.^22^ They may also suffer from high autofluorescence,^23^ or require irradiation conditions that result in excitation phototoxicity or thermally damage tissue.^24,25^ Consequently, there is a continuing need to develop new fluorescent probes with improved reporting characteristics. Here, we introduce a new pH-responsive probe that operates solely within the near infra-red (NIR) range and can report its dimensions using steady-state photoluminescence (PL) spectroscopy.

Responsive microgel particles (MGs)^26–32^ are crosslinked polymer colloid particles that swell in a good solvent or as the pH approaches the p*K*_a_ of the particle.^33^ MGs are appealing as probes due to their fast response, chemical tunability, biocompatibility, suitability for functionalisation, biodegradability and softness.^34–38^ MGs containing complementary fluorescent non-radiative energy transfer^39,40^ (NRET) fluorophores have been used for monitoring MG size and swelling.^41,42^ Fluorescent MGs can be prepared with high fluorescent stability, quantum yield and large Stokes shifts.^43–45^ In addition, the MGs can detect intracellular pH, temperature, and ion concentration changes.^46–48^ However, the PL intensity of many fluorophores can be greatly reduced or completely quenched when assembled into nanostructures due to the intermolecular π–π-stacking.^49–51^ Consequently, establishing new ratiometric probes with bright and stable emission is desirable.^52,53^

We recently investigated fluorescent probes that used short-wavelength excitation and emission.^7,41^ Short-wavelengths limit applications of such probes as a result of autofluorescence,^23^ photo-toxicity^54^ and poor light penetration depth.^55^ The probes were also weakly swelling with particle volume swelling ratios (*Q*) < 6. To overcome these limitations in this study we report *highly* swollen pH-responsive MG probes that contain complementary NIR fluorophores. NIR light is deeply penetrating in tissue. Moreover, NRET is highly sensitive to distance changes over the 1–10 nm range.^56^ NRET requires a pair of fluorophores wherein the donor emission overlaps with the acceptor absorption and energy transfer can occur *via* dipole-induced dipole coupling.^57^ The NIR fluorophores studied here have been used in *temperature-responsive* MGs based on poly(*N*-isopropylacrylamide) (PNP),^42,58^ but *not* in pH-responsive MGs.

Fluorescent MGs have attracted considerable attention.^59–62^ Liu *et al.*^60–62^ studied a ratiometric fluorescent MG labeled with nitrobenzoxadiazole (NBD) and rhodamine B pairs that reported the response to temperature, light, ions or sugar. Yang *et al.*^63^ synthesized a triaryl boron compound and Nile Red fluorescent MG probes and inserted them in NIH/3T3 cells for sensing temperature. Kim *et al.*^64^ also reported modulated multicolor fluorescent MGs labelled with NBD and spiropyran. Sung *et al.*^65^ embedded NIR cyanine dyes (Cy3 and Cy5) in biocompatible natural cationic pH-responsive MG with chitosan for imaging and NRET-based sensing in living cells. However, there are a lack of responsive MG systems that have both excitation and fluorescent emission within the deeply penetrating NIR region.^7,41^

**Scheme 1:**
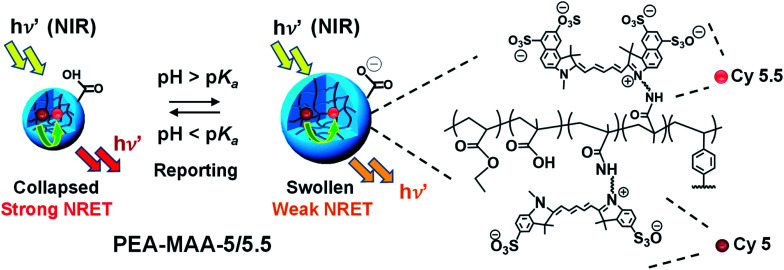
Depiction of reversible NRET fluorescent reporting within NIR PEA-MAA-5/5.5 MG probes in response to pH. The donor (Cy5) to acceptor (Cy5.5) distance increases as the MG swells which decreases the NRET efficiency and the emission blue-shifts.

The new NIR MG probes introduced here (Scheme 1) comprise poly(ethyl acrylate-*co*-methacrylic acid-*co*-divinylbenzene) (PEA-MAA-DVB) and the covalently linked, complementary, sulfo-cyanine flourophores Cy5 (donor) and Cy5.5 (acceptor). The MG probes are termed PEA-MAA-5/5.5. We selected the PEA-MAA-DVB MGs to attach the NRET fluorophores because previous work showed these MGs have very high pH-triggered swelling.^66^ The new PEA-MAA-5/5.5 MG probes introduced here have *four major advantages* compared to our previous probes: (1) they enable ratiometric PL intensity detection of particle swelling changes over much higher *Q* ranges (from 1–90). (2) Emission occurs when they are illuminated with NIR irradiation. (3) They emit in the NIR region. (4) The wavelength at maximum PL intensity (*λ*_max_) provides a *second* MG size-dependent parameter for reporting changes in MG swelling. These advantages and also the differences between the present study and our previous work are shown in Fig. S1 (ESI†).

Here, the new MG probes are first characterized and then the effects of pH variation on their PL spectra examined. These studies reveal that the maximum wavelength of the PL emission maximum provides a second new PL-based reporting mode for MG swelling. We then compare the PL reporting and colloidal stability of PEA-MAA-5/5.5 to a reference fluorescent NIR PNP MG system. This part of the study shows the advantages of our new pH-responsive MG. We also use the pH-responsive MG probes to demonstrate imaging both in stem cells and after sub-cutaneous injection in a tissue model. The new PEA-MAA-5/5.5 MGs have excellent future potential for remote NIR fluorescent reporting and imaging in physiological settings as well as high ionic strength environments such as waste water.

## Experimental section

### Materials

EA (99%), MAA (99%), DVB (80%), sodium dodecyl sulfate (SDS, 98.5%), ammonium persulfate (APS, 98%), 4-(4,6-dimethoxy-1,3,5-triazin-2-yl)-4-methylmorpholinium chloride (DMTMM, 96%), *N*-isopropylacrylamide (≥99%), *N*,*N*′-methylenebis(acrylamide) (MBAAm, 99%), *N*-(3-aminopropyl)methacrylamide hydrochloride (APMA), phosphate buffered saline (PBS), potassium dihydrogen phosphate (PDP, 98%), NaOH (97%), *N*,*N*′-dimethylformamide anhydrous (DMF, 99.8%) and AlamarBlue™ cell viability reagent were all purchased from Sigma-Aldrich. Sulfo-cyanine5 amine (Cy5-NH_2_), sulfo-cyanine5.5 amine (Cy5.5-NH_2_), sulfo-cyanine5 NHS ester (Cy5-NHS) and sulfo-cyanine5.5 NHS ester (Cy5.5-NHS) were obtained from Lumiprobe Ltd. Alexa Fluor 488 phalloidin and MesenPRO RS™ basal medium were obtained from Invitrogen Thermo Fisher Scientific. All materials were used as received. The water used was ultra-high purity and deionized.

### Synthesis of fluorescent NIR PEA-MAA-5/5.5 microgels

The precursor PEA-MAA-DVB MG was prepared by seed/starved-feed emulsion polymerization (see Scheme S1†). A mixed co-monomer solution (250 g) containing EA (164.4 g, 1.64 mol), MAA (82.2 g, 0.95 mol) and DVB (3.4 g, 0.026 mol) was prepared. Seed formation was conducted using a portion of the co-monomer mixture (31.5 g) after addition to water (517.5 g) containing SDS (1.8 g), K_2_HPO_4_ (3.15 g of 7.0 wt% solution) and APS (10.0 g of 2.0 wt% solution). The seed was formed at 80 °C with stirring under a nitrogen atmosphere over 30 min. The remaining co-monomer solution was added uniformly to the seed at a rate of 2.4 g min^−1^. After completion of the feed stage the temperature was maintained at 80 °C for 2.5 h. The MG dispersion was extensively dialyzed against water.

PEA-MAA-5/5.5 probe MGs were prepared as depicted in Scheme S2.† DMTMM (0.032 g, 0.116 mM) was added to the PEA-MAA-DVB MGs (0.10 mL, 18.3 wt%) dispersed in PDP (10 mL, pH 7.2) for 5 min. Then, Cy5-NH_2_ (0.105 mg, 0.141 μmol) and Cy5.5-NH_2_ (0.152 mg, 0.141 μmol) were dissolved in PDP buffer (10 mL, pH 7.8) and the solution added to the activated PEA-MAA-DVB dispersion and allowed to react for ∼24 h. The final pH was ∼7.5. The reaction mixture was purified by extensive dialysis against PDP (pH 8.5) and then water.

### Synthesis of PEA-MAA-5 and PEA-MAA-5.5 microgels

The methods used to synthesize non-NRET PEA-MAA-5 or PEA-MAA-5.5 particles were identical to that described above for PEA-MAA-5/5.5 (Scheme S2†). The only difference in procedure is that only one fluorophore was used for each system.

### Synthesis of PNP-5/5.5 microgel probes

The precursor PNP MG dispersion was prepared by precipitation polymerization.^58^ NIPAM (568 mg, 5.00 mmol), MBAAm (40.5 mg, 263 μmol), APMA (1.89 mg, 10.6 μmol) and SDS (23.9 mg) were dissolved in water (75 mL). This mixture was then deoxygenated with an N_2_ purge for 1 h at 70 °C in a 250 mL glass reactor, followed by the addition of aqueous APS solution (0.5 g, 4.0 μmol). The polymerization was conducted at 70 °C for 5 h under an N_2_ atmosphere. The dispersion was then cooled to room temperature and dialyzed extensively against water.

To prepare NIR excitation/emitting PNP-5/5.5 probe particles (Scheme S3†), PNP dispersion (4.0 mL, 0.80 wt%) was added to 8.0 mL PDP buffer (pH 7.1). Then, Cy5-NHS solution in DMF (0.216 mg, 0.278 μmol) and Cy5.5-NHS solution in DMF (0.246 mg, 0.278 μmol) were added and allowed to react for 24 h at room temperature. The reaction mixture was extensively purified by dialysis against water.

### Physical measurements

Titration measurements were conducted in the presence of aqueous NaCl (0.10 M) at room temperature with aqueous NaOH solution (0.10 M) and a Mettler Toledo DL15 instrument. The *z*-average particle size (*d*_*z*_) was determined by dynamic light scattering (DLS) using a Malvern Zetasizer Nano ZS. All measurements were conducted at 25 °C unless otherwise stated. The particle volume swelling ratio (*Q*) is calculated from the ratio of the swollen to collapsed particle volumes using the respective *d*_*z*_ values. Zeta potential measurements were also obtained using the Malvern Zetasizer Nano-ZS instrument. TEM images were obtained using a FEI Tecnai 12 BioTwin instrument operating at an accelerating voltage of 110 kV. The particles were stained using 1% uranyl acetate solution. Confocal laser scanning microscopy images (CLSM) images were obtained using a broadband confocal Leica TCS SP5 microscope. UV-visible absorption spectra were recorded with a Hitachi U-1800 UV spectrophotometer with a scan rate of 480 nm min^−1^. PL spectra were obtained using an Edinburgh Instruments FLS980 spectrometer. The position of the wavelength at maximum intensity (*λ*_max_) was determined by fitting the main peak to a 6^th^ order polynomial. Unless otherwise stated the excitation wavelength (*λ*_ex_) was 650 nm. Details concerning the reversibility studies, MG uptake by the cells and cytotoxicity assays are given in the ESI.†

### NIR imaging

NIR imaging was conducted using a laser with an incident wavelength of 650 nm. The sample investigated consisted of chicken breast (lean chicken breast purchased from Sainsbury Ltd., U.K.). A NIR camera (DCC 1240C from Thorlabs Ltd) fitted with a filter which blocked light below 715 nm was used to obtain digital NIR photographs of the samples. A PEA-MAA-5/5.5 dispersion (0.50 mL, 0.050 wt%) was injected below the surface of the chicken breast at room temperature using a 21-gauge needle.

## Results and discussion

### PEA-MAA-5/5.5 microgel probe characterization

**Fig. 1:**
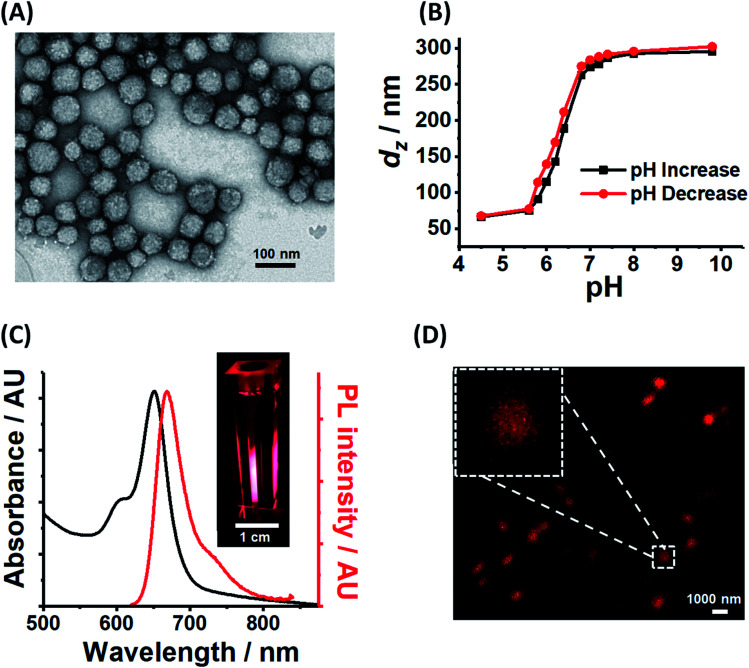
(A) TEM image for PEA-MAA-5/5.5. (B) Variation of *z*-average diameter (*d*_*z*_) for PEA-MAA-5/5.5 with pH. (C) UV-visible and photoluminescence (PL) spectra for PEA-MAA-5/5.5 at pH 5.8 (*λ*_ex_ = 600 nm). The inset shows the dispersions irradiated with 600 nm light. The camera was equipped with a filter that cut-off light below 645 nm. (D) CLSM image for PEA-MAA-5/5.5 dispersion (*λ*_ex_ = 633 nm) obtained at pH 7.4.

PEA-MAA-5/5.5 construction was performed in two steps. Firstly, PEA-MAA-DVB MG particles were synthesized by emulsion polymerization (Scheme S1, ESI†). The composition for the latter was PEA_0.58_-MAA_0.41_-DVB_0.01_ based on titration data (for MAA content, Fig. S2A, ESI†) and the assumption that all of the DVB added was incorporated. Then the fluorophores Cy5-NH_2_ and Cy5.5-NH_2_ (ref. 67) were covalently linked to the MG COO^−^ groups at pH 7.5 using DMTMM activation^68^ (Scheme S2, ESI†). The MAA content and apparent p*K*_a_ for PEA-MAA-5/5.5 estimated from potentiometric titration data (Table S1 (ESI†)) are 41.1 mol% and 6.5, respectively. The PEA-MAA-5/5.5 diameter from TEM is 57 nm (Fig. 1A). DLS data (Fig. 1B and S2B (ESI†)) gave a *z*-average diameter (*d*_*z*_) of 66 nm in the collapsed state (pH 4.5). At pH 7.4 the particles have a *d*_*z*_ value of 287 nm and are highly swollen (with *Q* = 82) due to deprotonation of the RCOOH groups. (Size polydispersity index data appear in Fig. S2D and DLS diameter distributions are shown in Fig. S3, ESI†). Fig. S1A (ESI†) shows that the *Q* values for PEA-MAA-5/5.5 are much higher than those for our earlier (non-NIR) probe particles.^7,41^ The zeta potential (*ζ*) was pH-responsive (Fig. S2C, ESI†) and decreased from −48 mV (at pH 7.4) to −29 mV (at pH 4.5). Hence, the anionic MGs have a high volume charge density in the swollen state, which is attributed to COO^−^ groups. Control MGs containing either Cy5 (PEA-MAA-5) or Cy5.5 (PEA-MAA-5.5) were also prepared and TEM images appear in Fig. S4 (ESI†). (PL and UV-visible spectra for the controls are shown in Fig. S5 (ESI†).) Composition and property data for all the systems prepared in this study are shown in Table S1 (ESI†).

We consider further the data relating to the pH-responsiveness of PEA-MAA-5/5.5. Comparison of the TEM image for the particles deposited at pH ∼ 5 (Fig. 1A) with the CLSM image of the particles swollen at pH 7.4 (Fig. 1D) provides clear evidence that a large pH-triggered size increase occurred. Moreover, the *d*_*z*_ and *ζ* values strongly increase with increasing pH and, interestingly, follow each other (see Fig. S2C, ESI†). Both increases begin after pH 4.5 and are complete at pH 8.0 (highlighted in Fig. S2C†). The *d*_*z*_ value originates from the whole particle;^69^ whereas, *ζ* is determined by the electrophoretic mobility which, in turn, is governed by the charge density at the particle periphery.^70^ Because *d*_*z*_ and *ζ* follow each other closely it follows that the pH-triggered swelling/de-swelling transitions for PEA-MAA-5/5.5 MG probes occur *uniformly* throughout the particles, *i.e.*, affine swelling occurs.

From the molar extinction coefficients measured for the fluorophores (Fig. S6, ESI†) and the UV-visible spectra for PEA-MAA-5/5.5 (Fig. 1C) the Cy5 and Cy5.5 contents in the MG were calculated as 0.010 mol% and 0.002 mol%, respectively. The relatively high concentration of Cy5 is attributed to differences in MG-fluorophore electrostatic interactions. The MG particles were negatively charged at pH 7.5; whereas, Cy5-NH_2_ and Cy5.5-NH_2_ had net charges of 0 and −2, respectively. (The structures of Cy5-NH_2_ and Cy5.5-NH_2_ are shown in Scheme S2 (ESI†).) Furthermore, the fluorophore contents for PEA-MAA-Cy5 and PEA-MAA-Cy5.5 were found to be 0.010 and 0.005 mol%, respectively. The relatively high concentration of Cy5 in the PEA-MAA-5/5.5 MGs together with the small separation of the two PL peaks (25 nm, Fig. S5A (ESI†)), resulted in one main PL peak in the PL spectra for PEA-MAA-Cy5/5.5 (Fig. 1C). We show below that the wavelength of this peak is dependent on *d*_*z*_. The PEA-MAA-5/5.5 dispersion emitted bright red light when illuminated at 600 nm (inset of Fig. 1C). A CLSM image of the MGs at pH 7.4 (Fig. 1D) confirms emission occurred in the red region of the electromagnetic spectrum.

### Reporting of pH-triggered microgel swelling

**Fig. 2:**
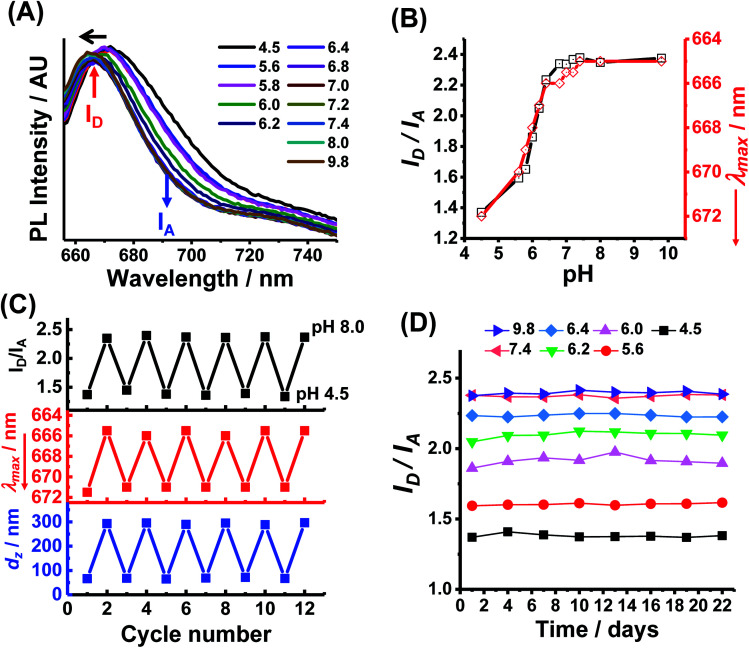
(A) PL spectra for PEA-MAA-5/5.5 MG dispersions at different pH values. The horizontal arrow shows the change with increasing pH. (B) Variation of the PL intensity ratio of the donor and acceptor peaks (*I*_D_/*I*_A_) and wavelength of maximum intensity (*λ*_max_) with pH obtained from the spectra shown in (A). The *λ*_max_ values are plotted in *reverse* order. (C) Reversibility experiments for *I*_D_/*I*_A_, *λ*_max_ and *d*_*z*_. (D) Measured *I*_D_/*I*_A_ values for PEA-MAA-5/5.5 stored at a range of pH values (shown) and at room temperature in the dark.

NRET is possible for PEA-MAA-5/5.5 MGs because there is substantial overlap of the main emission peak for Cy5 (667 nm) with the main absorption band of Cy5.5 (674 nm) (see Fig, S7, ESI†). We used the PL maxima for Cy5 in PEA-MAA-5 (667 nm) and Cy5.5 in PEA-MAA-5.5 (692 nm) (Fig. S5A, ESI†) for the donor intensity (*I*_D_) and acceptor intensity (*I*_A_), respectively, to analyze the PEA-MAA-5/5.5 MG spectra. Fig. 2A shows the effect of pH on the PL spectra. The wavelength of maximum intensity (*λ*_max_) blue-shifts as the pH increased. Both the (*I*_D_/*I*_A_) ratio and *λ*_max_ are very sensitive to pH-triggered swelling (see Fig. 2B). The *I*_D_/*I*_A_ ratio increased from 1.4 to 2.4 with increasing pH. Simultaneously, *λ*_max_ decreased from 672 to 665 nm. Both the excitation and emission wavelengths for PEA-MAA-5/5.5 occurred in the NIR region. The *λ*_max_ values (Fig. 2B) mostly follow the *I*_D_/*I*_A_ values as the pH increased. The *I*_D_/*I*_A_ values increase and *λ*_max_ blue-shift occur because energy transfer is less efficient as the inter-fluorophore separation increases.^71^ These changes follow the pH-triggered changes of the *d*_*z*_ values in Fig. 1B. The *I*_D_/*I*_A_ and *λ*_max_ values are reported over a very wide *Q* range (*i.e.*, 1–90) as shown in Fig. S8 (ESI†). Hence, *P*EA-MAA-Cy5/5.5 MG has two PL modes for reporting swelling, *i.e.*, *I*_D_/*I*_A_ and *λ*_max_ modes.

Multiple-run experiments for *I*_D_/*I*_A_, *λ*_max_ and *d*_*z*_ of PEA-MAA-5/5.5 using pH cycling between 4.5 and 8.0 were conducted to investigate reversibility (Fig. 2C). The results show negligible drift and, hence, good reversibility for all the parameters. This is attributable to the high zeta potentials for PEA-MAA-5/5.5 (Fig. S2C, ESI†) which help prevent aggregation. Dispersion stability during storage is also important for applications. The *I*_D_/*I*_A_ ratio (Fig. 2D), *λ*_max_ data (Fig. S9A, ESI†) and *d*_*z*_ data (Fig. S9B, ESI†) for PEA-MAA-5/5.5 MGs were measured at pH values in the range of 4.5–9.8 for 22 days. These values changed by an average of less than 5% and so PEA-MAA-5/5/5 MG had good stability.

### Comparing reporting from pH-responsive and temperature-responsive NIR microgels

**Fig. 3:**
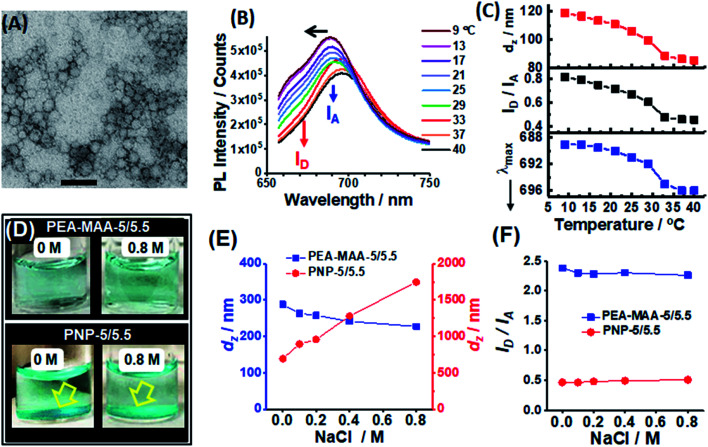
(A) TEM image of poly(*N*-isopropylacrylamide)-based NIR MGs (PNP-5/5.5). Scale bar: 100 nm. (B) PL spectra for PNP-5/5.5 measured at different temperatures. (C) Variations of *d*_*z*_, *I*_D_/*I*_A_ and *λ*_max_ with temperature. The data from (B) and (C) were obtained in pure water. (D) Digital photographs of PEA-MAA-5/5.5 (top row) and PNP-5/5.5 (bottom row) dispersions in the absence and presence of NaCl (0.8 M). The dispersions also contained pH 7.4 buffer (0.10 M) and the temperature was 37 °C. The yellow arrows highlight aggregation. (E) Variation of *d*_*z*_ for both MGs with NaCl concentration. (F) Effect of NaCl concentration on the *I*_D_/*I*_A_ values. The data in (E) and (F) were measured at 37 °C.

We next compare the reporting properties of PEA-MAA-5/5.5 to an established temperature-responsive poly(*N*-isopropylacrylamide)-based NIR MG containing Cy5 and Cy5.5.^58^ The latter is denoted as PNP-5/5.5 and was prepared by precipitation polymerization (Scheme S3, ESI†). The particles are spherical with an average TEM diameter of 40 nm (Fig. 3A). The PNP-5/5.5 MG particles contained ∼0.020 mol% Cy5 and 0.050 mol% Cy5.5 using the UV-visible spectrum (Fig. S10, ESI†) and the molar extinction coefficients for the NHS-functionalized fluorophores (Fig. S11, ESI†). The PL maximum for PNP-5/5.5 (Fig. 3B) is dominated by Cy5.5. The relatively high Cy5.5 concentration in the PNP-5/5.5 MGs may be due to greater electrostatic attraction during coupling to the –NH_3_^+^ groups of the MGs for Cy5.5-NHS (charge = −3) compared to Cy5-NHS (charge = −1). The coupling reaction and Cy5.5-NHS and Cy5-NHS structures are shown in Scheme S3 (ESI†).

Fig. 3B shows the PL spectra change considerably as the temperature approaches the volume phase transition temperature (VPTT). The latter is ∼32 °C based on the temperature-dependence for *d*_*z*_ (see Fig. 3C, top), which agrees with the literature.^72^ The PNP-5/5.5 MG de-swelling is due to temperature-triggered disruption of the hydrogen bonding of water with the amide groups. The *I*_D_/*I*_A_ ratio for PNP-5/5.5 decreases from 0.8 to 0.4 when the temperature is increased from 9 to 40 °C (Fig. 3C, middle) due to increased NRET and mirrors the *d*_*z*_ changes. These data confirm that PNP-5/5.5 is also able to report changes of *d*_*z*_ in pure water.^58^ The *λ*_max_ value can also be used to report temperature-triggered de-swelling as shown in the bottom graph of Fig. 3C. Based on these results *t*he ability to use both *I*_D_/*I*_A_ and *λ*_max_ to report particle swelling appears to be general for MGs containing Cy5 and Cy5.5.

To test the ability of the MG probes to report swelling in the presence of electrolyte a series of PEA-MAA-5/5.5 and PNP-5/5.5 dispersions were prepared in pH 7.4 buffer (0.10 M) at 37 °C in the presence of various added NaCl concentrations. Visual inspection (Fig. 3D and S12, ESI†) showed that the PEA-MAA-5/5.5 dispersion did not have any aggregates in the presence of added NaCl – even at concentrations as high as 0.80 M! In contrast, at all NaCl concentrations (and even in the absence of added NaCl) a sedimented layer of aggregates was present for PNP-5/5.5. The *d*_*z*_ data (Fig. 3E) show that the PEA-MAA-5/5.5 particles slightly de-swelled with increasing NaCl concentration, which is due to electrostatic screening.^73^ In contrast, the *d*_*z*_ values for PNP-5/5.5 increased strongly with increasing NaCl concentration at 37 °C due to aggregation.

The different colloidal stabilities of the PEA-MAA-5/5.5 and PNP-5/5.5 dispersions to electrolyte are due to differences in the dispersion stabilisation mechanisms. Zeta potential (*ζ*) data measured for PEA-MAA-5/5.5 and PNP-5/5.5 (Fig. S13, ESI†) show that the former has much larger *ζ* values in the presence of NaCl than the latter. This difference is due to the high content of COO^−^ groups in PEA-MAA-5/5.5 at pH 7.4. Because the PNP-5/5.5 MGs collapsed at 37 °C, which is above their VPTT of 32 °C (above), they relied exclusively on electrostatic stabilization for colloidal stability. However, this was compromised in the presence of electrolyte concentrations greater than or equal to 0.10 M. In contrast the PEA-MAA-5/5.5 particles remained mostly swollen based on the *d*_*z*_ data (Fig. 3E). Hence, PEA-MAA-5/5.5 dispersions are stabilized by electrostatic and steric interactions, *i.e.*, they are *electrosterically* stabilized.^74^ Such strong stabilization imparted superior colloidal stability under all conditions studied. Indeed, the PEA-MAA-5/5.5 probe has colloidal stability at electrolyte concentrations at least 5 times higher than physiological ionic strength. The superior stability of PEA-MAA-5/5 originates from the very high MAA content (41 mol%) which provides high charge densities and robust particle swelling in electrolyte solutions.

The PL spectra for the two probes (Fig. S14A and B, ESI†), *I*_D_/*I*_A_ (Fig. 3F) and *λ*_max_ values (Fig. S14C, ESI†) show major differences. The *I*_D_/*I*_A_ and *λ*_max_ values for PEA-MAA-5/5.5 are close to their fully swollen values (from Fig. 2B) and decrease slightly with increasing NaCl concentration (Fig. 3F and S14C (ESI†)). These decreases are due to electrolyte induced de-swelling of the PEA-MAA-5/5.5 probe particles (Fig. 3E). In contrast all of the *I*_D_/*I*_A_ (Fig. 3F) and *λ*_max_ values (Fig. S14C, ESI†) for PNP-5/5.5 correspond to the collapsed state (from Fig. 3C). Whilst the PNP-5/5.5 PL data have correctly reported the swelling state, the system is compromised in terms of further reporting because its swelling state cannot change at physiological temperature. It is also aggregated. Hence, the PEA-MAA-5/5.5 NIR probe MG has the potential advantages over PNP-5/5.5 of being colloidally stable and in a swollen state (and able to report swelling changes) under physiological conditions and in high salt concentration environments. PNP-5/5.5 is best suited to very low electrolyte concentrations (<0.10 M) where it remains colloidally stable.

The loading and position of attachment of the fluorophores within these MG probes will affect NRET. If the loading of either fluorophore is too low then the average separation between the donor and acceptor will be much greater than the Förster distance (6.9 nm (ref. 75)) and NRET will no longer be observed. We used reaction solutions containing *both* fluorophores to ensure close attachment of each in the MG network. In this study, the mole ratios of Cy5 to Cy5.5 decreased from 5.0 for PEA-MAA-5/5.5 to 0.40 for PNP-5/5.5. The changes in *I*_D_/*I*_A_ and *λ*_max_ were successfully used to report diameter changes for both MG probe systems. Hence, we conclude that NRET will allow dual mode PL reporting for similar MG-5/5.5 probes provided they are prepared using the methods described this work. Accordingly, the mole ratio of Cy5 to Cy5.5 within the MG probes should be in the range 0.40 to 5.0 and their total (summed) concentration should be between 0.012 and 0.070 mol% (Table S1, ESI†).

### Imaging PEA-MAA-5/5.5 within stem cells and after subcutaneous injection

**Fig. 4:**
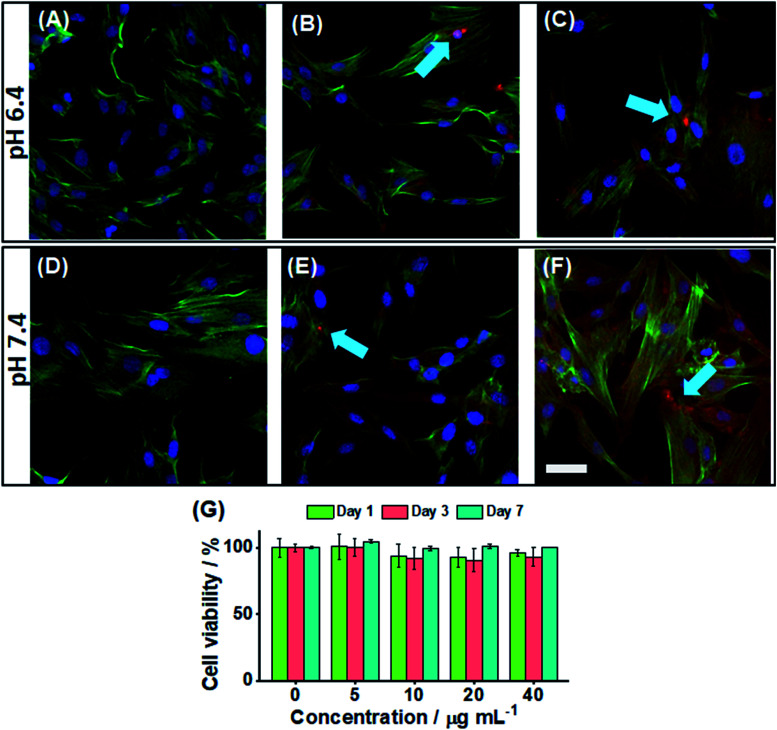
Merged CLSM images of stem cells at pH 6.4 (A–C) and 7.4 (D–F) with PEA-MAA-5/5.5 MG concentrations of (A and D) 0, (B and E) 20 μg mL^−1^ and (C and F) 40 μg mL^−1^. The colors correspond to wavelengths of 410–480 nm (blue), 490–550 nm (green) and 650–800 nm (red). The scale bar is 50 μm and applies to all images. The blue arrows highlight MGs. (G) Cell cytotoxicity data (*n* = 3) for stem cells at various MG concentrations at pH 7.4. The error bars are the standard deviation.

We investigated the ability to image the PEA-MAA-5/5.5 probes within adipose-derived stem cells. The pH values of 6.4 (Fig. 4A–C) and 7.4 (Fig. 4D–F) were investigated. The blue stain shows the nucleus, and the green stain highlights the actin, while the red color is from the PEA-MAA-5/5.5 MGs. These MG probes were able to cross the cell membrane^76,77^ of stem cells after only 4 h incubation. There was no need to use cationic^78^ or lipophilic^79^ transfection agents. This property is potentially useful because stem cells can be difficult to transfect.^80^ The regions of locally high MG concentration in Fig. 4B, C, E and F and are indicated with blue arrows. Additional images recorded using 0, 5, 10, 20 and 40 μg mL^−1^ are shown for each color channel and also bright field white light at pH 6.4 and 7.4, respectively, in Fig. S15 and S16 (ESI†). Cell imaging using white light showed normal cell growth morphology indicating that PEA-MAA-5/5.5 produced little toxicity inside the cells (see bright field images in Fig. S15 and S16 (ESI†)). We measured the fluorescence intensity of a series of MG dispersions with the same concentrations as used for stem cell uptake *in vitro* using PL spectroscopy (see Fig. S17, ESI†). The *I*_D_ and *I*_A_ values are linear with PEA-MAA-5/5.5 concentration implying facile tuning of the PL intensity (Fig. S17B, ESI†). The *I*_D_/*I*_A_ ratios and *λ*_max_ values were not affected by probe concentration (Fig. S17C, ESI†), confirming good probe stability.

*In vitro* cytotoxicity of the PEA-MAA-5/5.5 probes was measured *via* the Alarmar blue™ assay using adipose-derived stem cells. The cells were incubated with PEA-MAA-5/5.5 at concentrations ranging from 5 to 40 μg mL^−1^ for 1, 3, and 7 days. The cells proliferated in the first three days, and then the cell viability stayed the same or increased for the next four days. The final viability after 7 days was over 95% (see Fig. 4G). There was not any significant cytotoxicity of PEA-MAA-5/5.5 for the stem cells under the conditions employed.

**Fig. 5:**
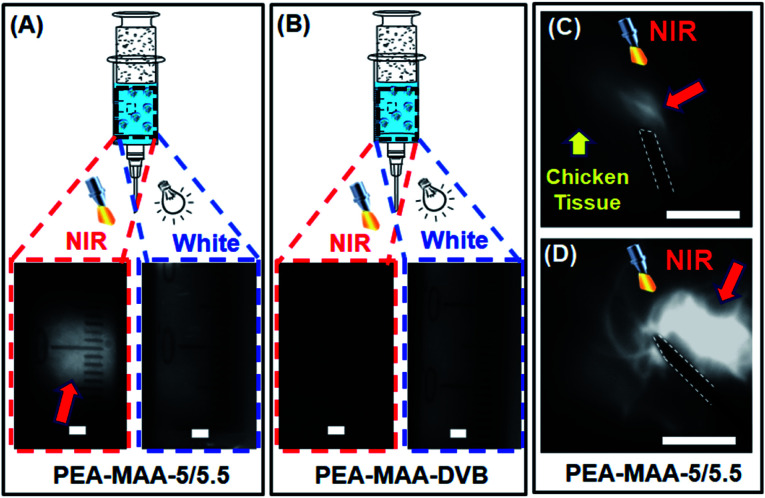
Photographs of (A) fluorescent PEA-MAA-5/5.5 and (B) non-fluorescent PEA-MAA-DVB MG dispersions in syringe barrels. The images were captured with an NIR camera using illumination with NIR light (left hand side) and white light (right hand side). Scale bars: 0.2 cm. NIR images of the (C) initial and (D) the end stages of the subcutaneous injection of the PEA-MAA-5/5.5 dispersion into chicken breast. The red arrows highlight NIR emission. The white outlines show the needle. The injection depth was 5.0 mm. Scale bars: 1 cm.

We investigated the ability to use PEA-MAA-5/5.5 as an injectable MG for NIR imaging. Fig. 5A and B show, respectively, images for PEA-MAA-5/5.5 probe and the parent non-fluorescent PEA-MAA-DVB MG control in syringe barrels illuminated by NIR and white light. The PEA-MAA-5/5.5 dispersion appears white when illuminated with NIR light (650 nm) and imaged with an NIR camera (Fig. 5A, left hand side). In contrast PEA-MAA-DVB does not emit NIR light and the dispersion is dark when illuminated with NIR light (Fig. 5B, LHS). Also, both PEA-MAA-5/5.5 and PEA-MAA-DVB do not emit NIR light when illuminated with white light (Fig. 5A and B, RHS). These experiments demonstrate that only PEA-MAA-5/5.5 can be imaged using NIR when illuminated with NIR light. We next investigated the imaging ability for subcutaneous injection of PEA-MAA-5/5.5 into chicken tissue (see Fig. 5C and D). The PEA-MAA-5/5.5 probe was injected at a depth of 5.0 mm within the chicken breast. (That latter tissue is dark under NIR irradiation.) NIR emission appeared (Fig. 5C) and gradually spread (Fig. 5D) as the injection proceeded. Hence, PEA-MAA-5/5.5 can be imaged by NIR light when subcutaneously injected in such tissue.

**Fig. 6:**
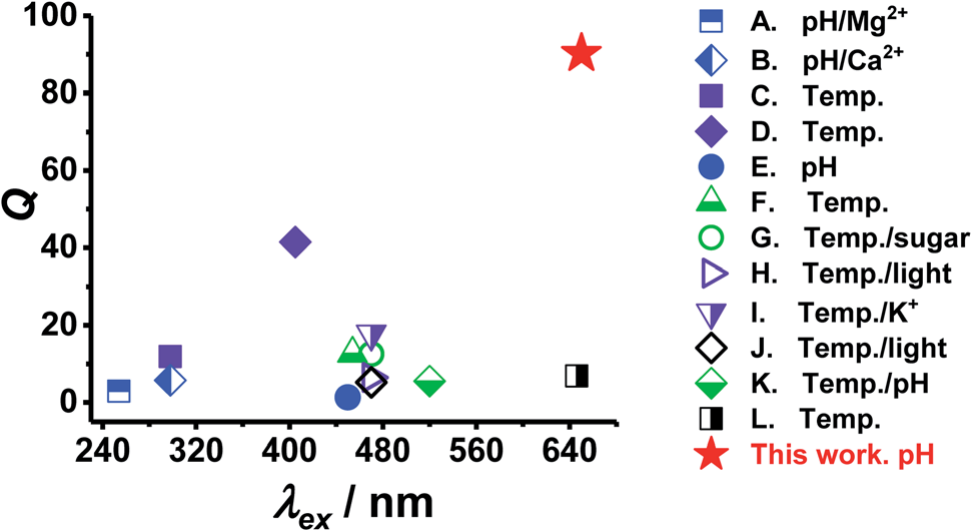
Comparison of the properties of PEA-MAA-5/5.5 MGs with other ratiometric fluorescent stimuli-responsive swollen probes that use NRET. The particle volume swelling ratios (*Q*) and excitation wavelengths (*λ*_ex_) used appear in Table S2 (ESI†).

We compare the performance of PEA-MAA-5/5.5 in terms of particle swelling ratio (*Q*) and *λ*_ex_ with other reported stimuli-responsive ratiometric NRET-based fluorescent nanoscale probes in Fig. 6. *Q* is the ratio of swollen to collapsed particle volume and was calculated using the published DLS data. (The data used are shown in Table S2, ESI†). The values for *λ*_ex_ were given in the publications. The probes were responsive to pH, metal cations, pH, sugar or light and are indicated. PEA-MAA-5/5.5 (red star) has by far the highest *Q* value of all of these systems. Furthermore, PEA-MAA-5/5.5 can be excited using a very high *λ*_ex_. It also emits at a relatively high wavelength (*λ*_em_) compared to the other systems. The main competitor in terms of *λ*_ex_ is the temperature-responsive PNP-based system (L in Fig. 6). However, that *Q* value is more than a factor of 10 lower than that for PEA-MAA-5/5.5. That system was synthesized in this study (PNP-5/5.5) and was unstable to electrolyte (*cf.* PEA-MAA-5/5.5) as discussed above (Fig. 3). Unlike most systems in Fig. 6, PEA-MAA-5/5.5 is pH-responsive (Fig. 2B) and, due to electrosteric stabilization, is only weakly affected by ionic strength (Fig. 3E and F). Hence, PEA-MAA-5/5.5 has these unique advantages, as well as dual-mode PL reporting, offering potential as a pH-responsive NIR probe for reporting particle swelling.

We envisage potential use for PEA-MAA-5/5.5 as strain reporting probes for gel-based implants.^81^ For example injectable gels have been used for increasing the height of degenerated intervertebral discs.^82^ Inclusion of our NIR PEA-MAA-5/5.5 probes into such gels would, in principle, enable the *d*_*z*_ value (and strain) of the MGs to be reported externally *via* NIR emission using *I*_D_/*I*_A_ and/or *λ*_max_. Such reporting should also be possible if the PEA-MAA-5/5.5 probes were internalized in cells within soft tissue. To test the ability to estimate *d*_*z*_ for our probes using *I*_D_/*I*_A_ and/or *λ*_max_ we constructed calibration curves (Fig. S18A and B, ESI†) using these parameters and the respective measured *d*_*z*_ values from Fig. 1B and 2B. We then used the calibration curves to calculate *d*_*z*_ values for PEA-MAA-5/5.5 from *I*_D_/*I*_A_ and/or *λ*_max_ data in the later parts of this study, *i.e.*, Fig. 2C, D, S9 (ESI†) and 3E. These *calculated d*_*z*_ values are plotted against the *measured* values in Fig. S18C (ESI†). Good agreement between the calculated and measured values is evident using *I*_D_/*I*_A_, or *λ*_max_ as well as the average of the *d*_*z*_ values calculated from *I*_D_/*I*_A_ and *λ*_max_. Whilst this analysis shows that all our results are self-consistent it also paves the way for future work using such NIR probes in cells and implants to monitor internal strain remotely.

## Conclusions

In this work we have studied new PEA-MAA-5/5.5 pH-responsive NIR MG probes. The PEA-MAA-5/5.5 MGs can report pH-triggered swelling using NIR fluorescence reversibly in a range of conditions using both ratiometric intensity as well as *λ*_max_. The PEA-MAA-5/5.5 probes have excellent colloidal and fluorescent signal stability at physiological pH, ionic strength and temperature. These new probes, which benefit from electrosteric stabilization, have better colloidal stability than the reference PNP-5/5.5 MGs. We have shown that *λ*_max_ provides a second PL addressable mode that is able to report swelling changes for both PEA-MAA-5/5.5 and PNP-5/5.5 MGs. This unexpected and general result is attributed to the incomplete separation of the PL maxima due to the acceptor and donor fluorophores used. PEA-MAA-5/5.5 MGs are not cytotoxic to adipose stem cells and can potentially be used for NIR cell imaging. The results from subcutaneous injection study indicate that the PEA-MAA-5/5.5 also has potential for NIR imaging in tissue. Because the PL signal is very sensitive to MG swelling and NIR is deeply penetrating the PEA-MAA-5/5.5 MGs could be used to report pH or swelling changes inside cells or other complex environments such as tissue or synthetic gels. Furthermore, the total concentration of Cy5 and Cy5.5 used to obtain NIR reporting and imaging in this study was only ∼5 × 10^−7^ M to 5 × 10^−6^ M and PEA-MAA-5/5.5 had good stability (Fig. 2D). Hence, these new probes potentially provide a versatile and cost-effective alternative for NIR reporting and imaging. The PEA-MAA-5/5.5 MG system also has excellent potential to be used for environmental monitoring of aqueous solutions with high electrolyte concentration (*e.g.*, waste water).

## Conflicts of interest

There are no conflicts of interest to declare.

## Supplementary Material

NA-002-D0NA00581A-s001
