# Supplementary material for: Highly swelling pH-responsive microgels for dual mode near infra-red fluorescence reporting and imaging
Source: Nanoscale Adv. 2020 Aug 14;2(9):4261–71. doi: 10.1039/d0na00581a (PMC9419105; doi:10.1039/d0na00581a)
Supplement: NA-002-D0NA00581A-s001 [file NA-002-D0NA00581A-s001.pdf]

Electronic Supplementary Material (ESI) for Nanoscale Advances.
This journal is © The Royal Society of Chemistry 2020

Supporting Information for

Highly swelling pH-responsive microgels for dual mode near infra-red fluorescence reporting and imaging

Mingning Zhu[a,]*, Dongdong Lu[a,]*, Qing Lian[a], Shanglin Wu[a], Wenkai Wang[a], L. Andrew Lyon[b,c], Weiguang Wang[d], Paulo Bártolo[d], Mark Dickinson[e] and Brian R. Saunders[a,]*

[a]*Department of Materials, University of Manchester, MSS Tower, Manchester, M13 9PL, U.K.*

[b]*Schmid College of Science and Technology, Chapman University, Orange, CA, 92866, USA.*

[c]*Fowler School of Engineering, Chapman University, Orange, CA, 92866, USA.*

[d]*Department of Mechanical, Aerospace and Civil Engineering, School of Engineering, Faculty of Science and Engineering, University of Manchester, Manchester M13 9PL, U.K.*

[e]*Photon Science Institute, University of Manchester, Oxford Road, Manchester, M13 9PL, U.K.*

ADDITIONAL EXPERIMENTAL DETAILS

Reversibility and cycling studies for microgel probes in dispersion

To test swelling and reversibility, PEA-MAA-5/5.5 microgel (MG) probes were dispersed in pH 4.5 or pH 9.8 buffer. For the cycling pH tests, the PEA-MAA-5/5.5 dispersion in PDP buffer (pH 4.5, 1.0 mM) was placed in dialysis tubing (68 kDa MWCO, Fisher Scientific) and this was placed in a relatively large amount of water to remove buffer ions (10 L). Then,

the sample was placed in a much greater volume of buffer solution which was periodically switched from pH 4.5 to 8.0. After each pH change, the dispersion was dialyzed in water and the next buffer solution. A period of 24 h was allowed for the internal pH to equilibrate.

Microgel uptake by cells

In vitro biological studies were conducted using human adipose-derived stem cells (STEMPRO®, Invitrogen, USA) ranging from passage 7 to 9. Cells were cultured in T75 cell culture flasks (Sigma-Aldrich) with MesenPRO RS™ Basal Media (Invitrogen, USA) until 80% confluency and harvested by the use of a 0.05% trypsin solution (Invitrogen, USA). Cells in medium (3.0×10^4 in 150 µL) were then seeded on each well of a 24-well plate. Each well contained 310 µL medium at pH 7.4. PEA-MAA-5/5.5 probe dispersion (40 µL) was added to each well and carefully mixed. The final concentration of probe was 5, 10, 20, 40 µg/mL respectively. Incubation was conducted for 4 h at 5% CO_2, 95% humidity and 37 °C. After incubation, the plates were washed thoroughly with sterile PBS and fixed with 10% (w/v) neutral buffered formalin (Sigma-Aldrich) for 30 min at room temperature. Subsequently, samples were rinsed three times with PBS for the removal of formalin, made permeable with 0.5 mL 0.1 % Triton-X100 (Sigma-Aldrich) in PBS at room temperature for 10 min and then rinsed three times for the removal of Triton-X100. Afterwards, FBS solution (500 µL of 8% solution) was added into each sample and incubated for 60 min at room temperature to block non-specific binding. The cells were stained with Alexa Fluor 488 phalloidin at the manufacturer recommended concentration for cell actin protein for 45 min at room temperature and were washed rapidly three times with PBS. Finally, samples were left in the nuclei staining solution 4′,6-diamidine-2′-phenylindole dihydrochloride (DAPI) for 10 min prior to removal and rinsed twice thoroughly with PBS. Images for the MG probe uptake experiments were obtained with a Leica TCS SP5 confocal laser scanning microscope

(λ_{ex} = 633 nm). Images of the MG probe (PEA-MAA-5/5.5, λ_{ex} = 633 nm), green-emitting cellular organelles (Alexa Fluor 488, λ_{ex} = 488 nm) and blue-emitting cell nucleus (DAPI, λ_{ex} = 405 nm) were acquired in optical windows between 410 - 480 nm, 490 - 550 nm and 650 - 800 nm, respectively.

Cytotoxicity assays

Human adipose-derived stem cell viability / proliferation tests in the presence of PEA-MAA-5/5.5 probe dispersions were measured using an AlamarBlue™ assay at 1, 3 and 7 days. The cells were seeded on 24-well cell culture plates with Gibco™ MesenPRO RS™ Basal Medium with a density of appropriately 3.0×10^4 cells per well and grown overnight. After removal of the original medium in each well, 500 μL of Basal medium containing predetermined concentrations of PEA-MAA-5/5.5 at pH 7.4 was added to each well. A MG-free cell group was used as control. The cell-seeded dispersion was incubated at standard conditions (37 °C under 5% CO_2 and 95% humidity). At each time point cell culture media was removed and AlamarBlue™ assay in PBS solution (0.5 mL, 0.010 mg/ml) was added to each well and incubated for 4 h under standard conditions. Finally, 150 μL of each sample solution was transferred to a 96-well plate. The cell viabilities were determined using fluorescence (λ_{ex} = 540 nm) with an emission wavelength of 590 nm using a Synergy™ HT (BioTek, USA). Experiments were performed three times for each time point and concentration. The cell viability was calculated using the following equation[1]:

$$\text{Cell viability (\%)} = \frac{I_{\text{Sample}}}{I_{\text{Control}}} \times 100\% \qquad (1)$$

where I_{sample} and I_{control} are the intensity of sample and control wells, respectively.

SCHEMES

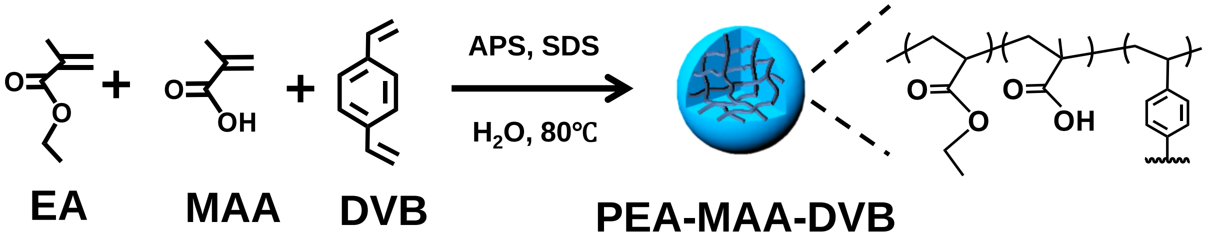

Scheme S1. Depiction of the synthesis of PEA-MAA-DVB MG.

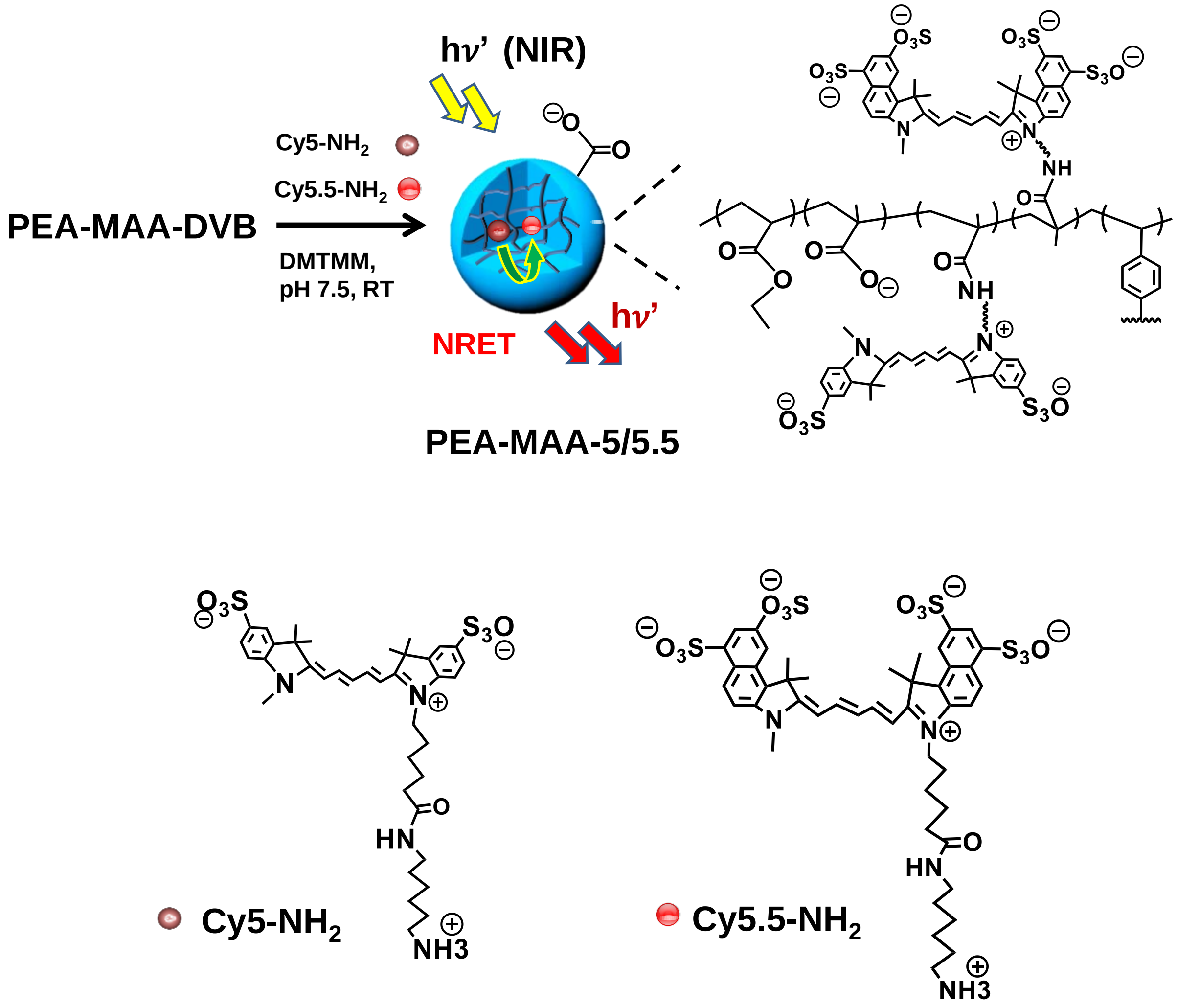

Scheme S2. Depiction of the synthesis of fluorescent near infra-red (NIR) PEA-MAA-5/5.5 MGs. The same method was used to synthesise PEA-MAA-5 and PEA-MAA-5.5 MGs using only Cy5-NH_2 or Cy5.5-NH_2, respectively. The structures of sulfo-cyanine5 amine (Cy5-NH_2) and sulfo-cyanine5.5 amine (Cy5.5-NH_2) are shown. The PEA-MAA-5/5.5 MGs report pH-triggered size changes using non-radiative energy transfer (NRET).

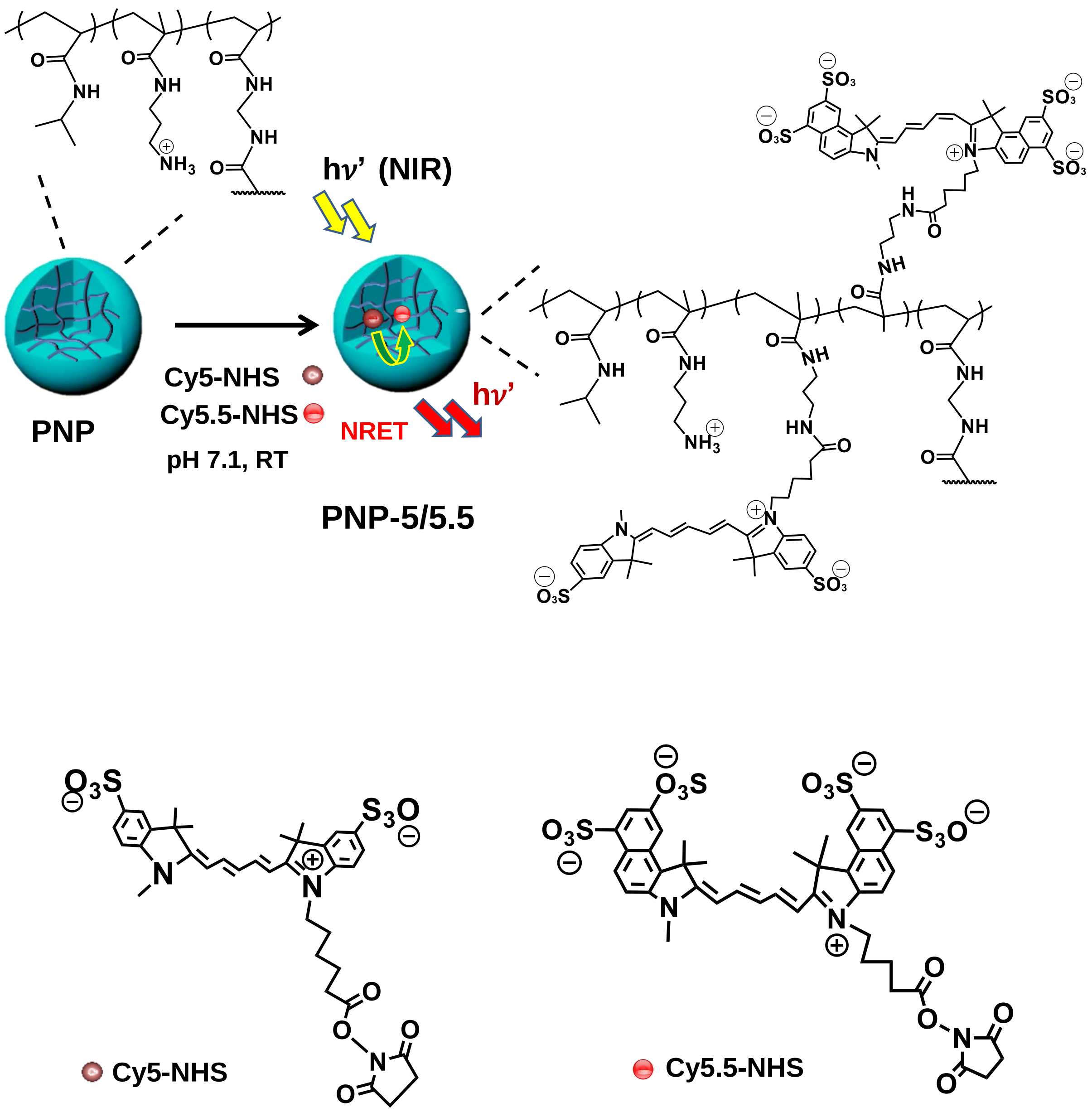

Scheme S3. Synthesis of temperature-responsive NIR poly(*N*-isopropylacrylamide)-based MG probe particles (PNP-5/5.5). The structures of Cy5-NHS and Cy5.5-NHS are shown. The PNP-5/5.5 MGs are used in this study as a reference system that reports temperature-triggered size changes using NRET[2].

FIGURES

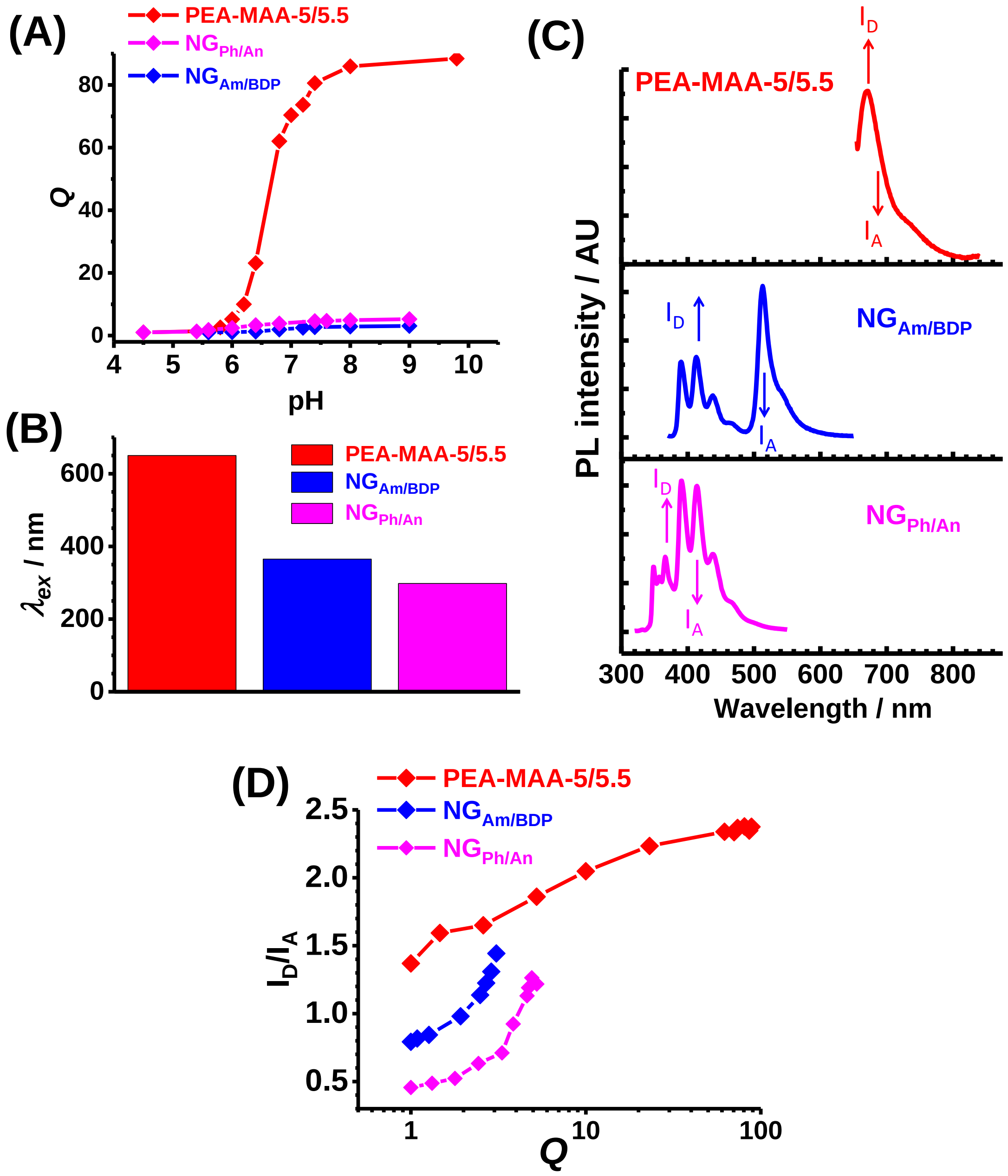

Figure S1. Comparison of the new PEA-MAA-5/5.5 MG probe properties (data shown in red) used in this study with nanogel probes ($NG_{AM/BDP}$ and $NG_{Ph/An}$) from earlier work[3, 4]. The variation of particle volume swelling ratio (Q) with pH is shown in **(A)**. **(B)** Comparison of the excitation wavelengths. The PL spectra of the probes are shown in **(C)** and the changes of the donor (I_D) and acceptor (I_A) intensities with increasing pH. **(D)** Variation of I_D/I_A with Q. The PEA-MAA-5/5.5 probes use NIR excitation and emit in the NIR and provide I_D/I_A data over a much wider Q range (1 – 90) than the previous pH-responsive probes.

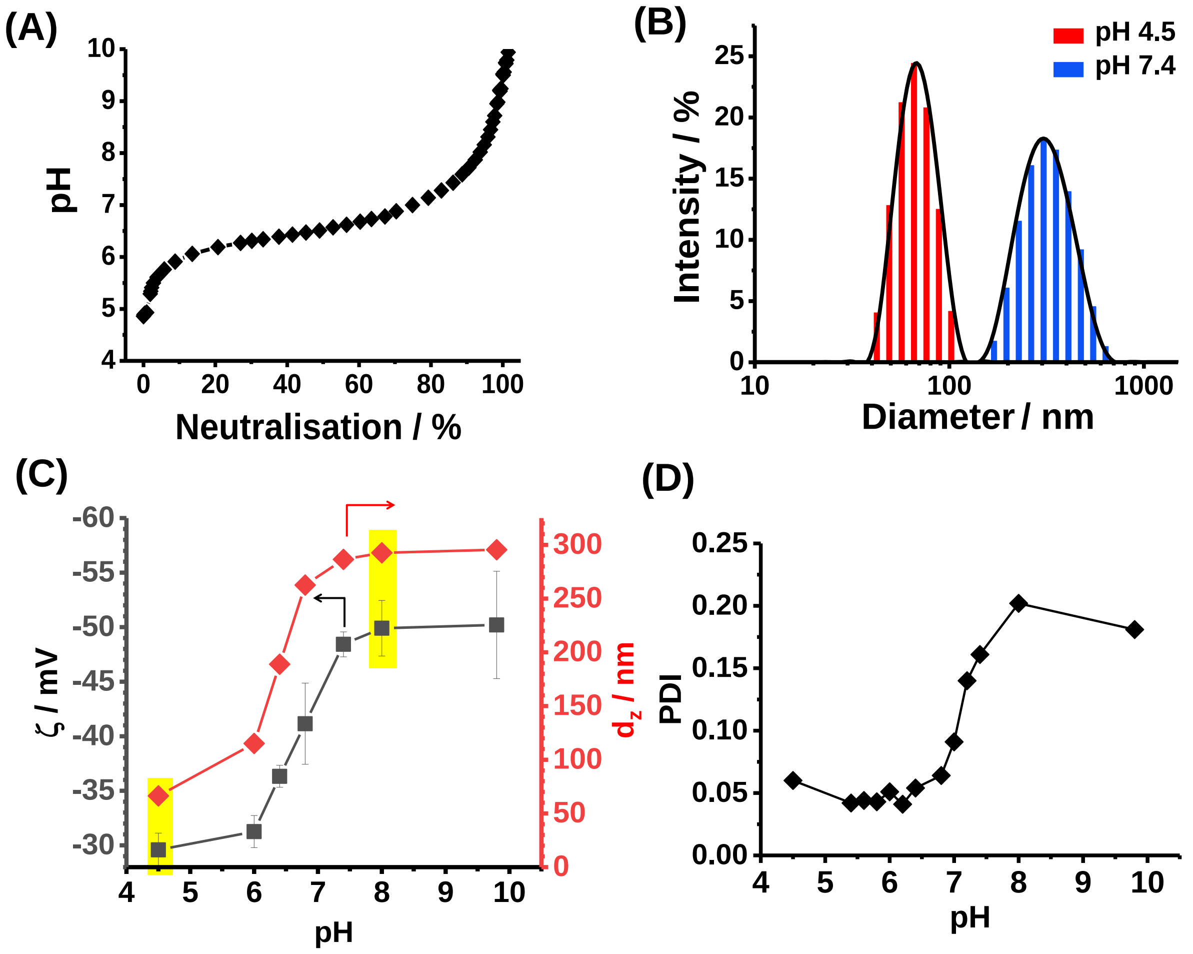

Figure S2. (**A**) Potentiometric titration data for PEA-MAA-DVB dispersion. The apparent pK_a was obtained from the pH at 50% neutralisation. (**B**) DLS diameter distribution for PEA-MAA-5/5.5 at a pH value of 4.5 and 7.4. (**C**) Zeta potential data measured as a function of pH for PEA-MAA-5/5.5. The error bars for the latter are standard deviations (n = 5). The d_z data are also shown for comparison. The yellow regions in (C) highlight the data obtained at pH 4.5 and pH 8.0. **(D)** Shows the variation of PDI with pH for the d_z data from (C).

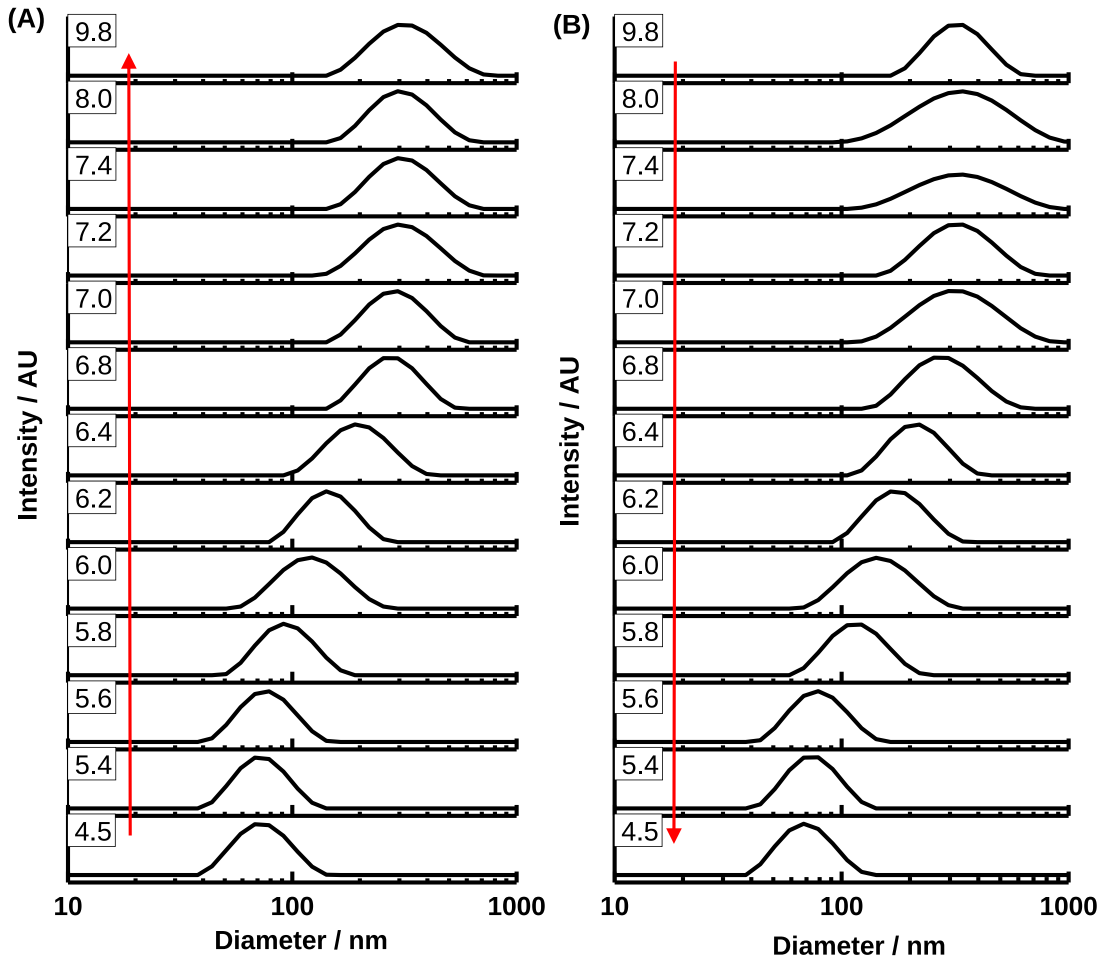

Figure S3. PEA-MAA-5/5.5 DLS diameter distributions from which the d_z values in Figure 1B were calculated. **(A)** is the increasing pH ramp and **(B)** is the decreasing pH ramp.

(A)

(B)

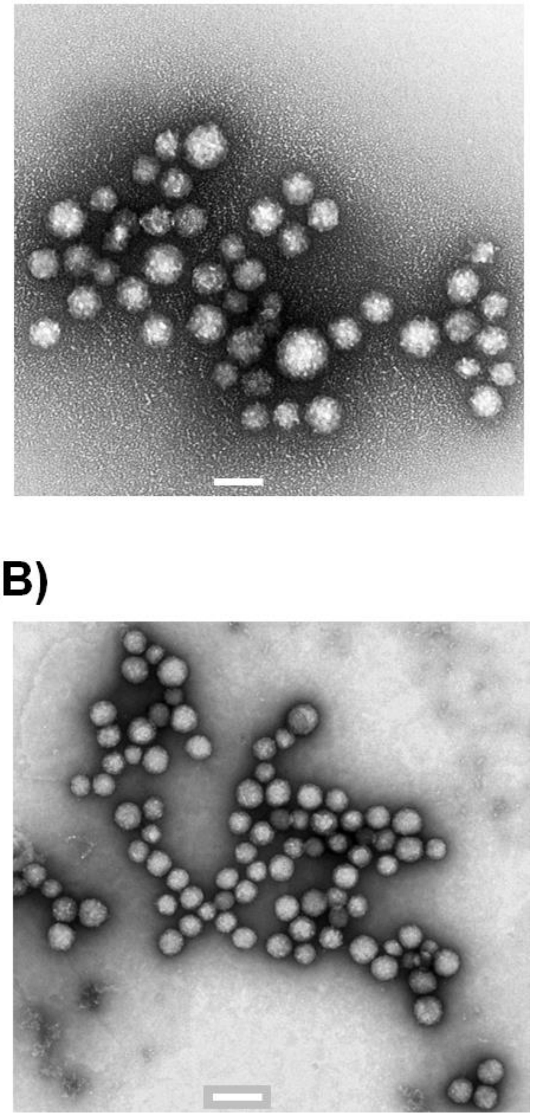

Figure S4. TEM images for **(A)** PEA-MAA-5 and **(B)** PEA-MAA-5.5. Scale bars: 100 nm.

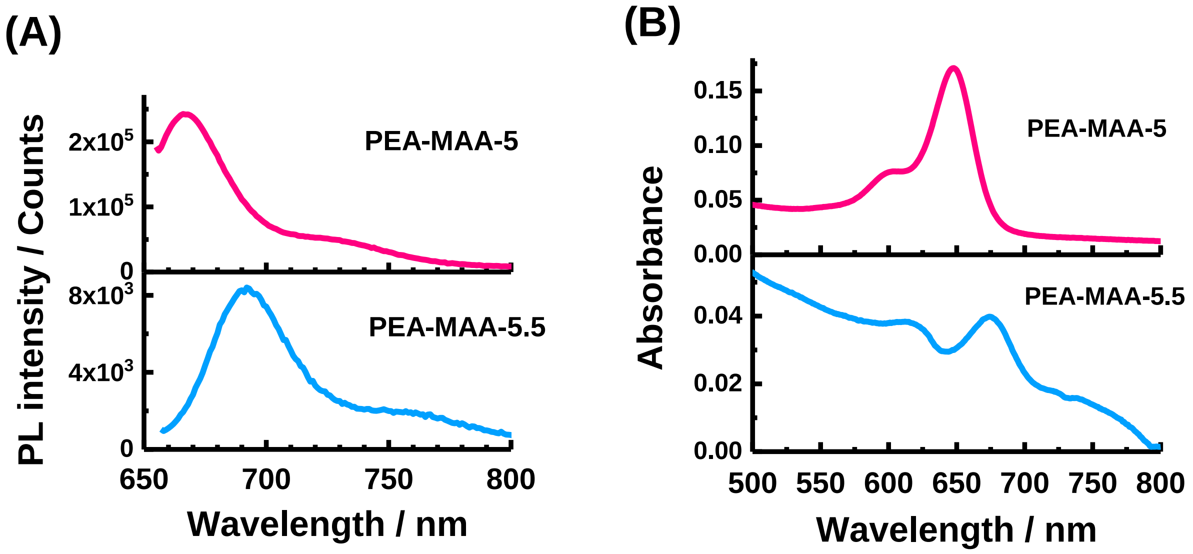

Figure S5. **(A)** Photoluminescence (PL) and **(B)** UV-visible spectra for PEA-MAA-5 and PEA-MAA-5.5.

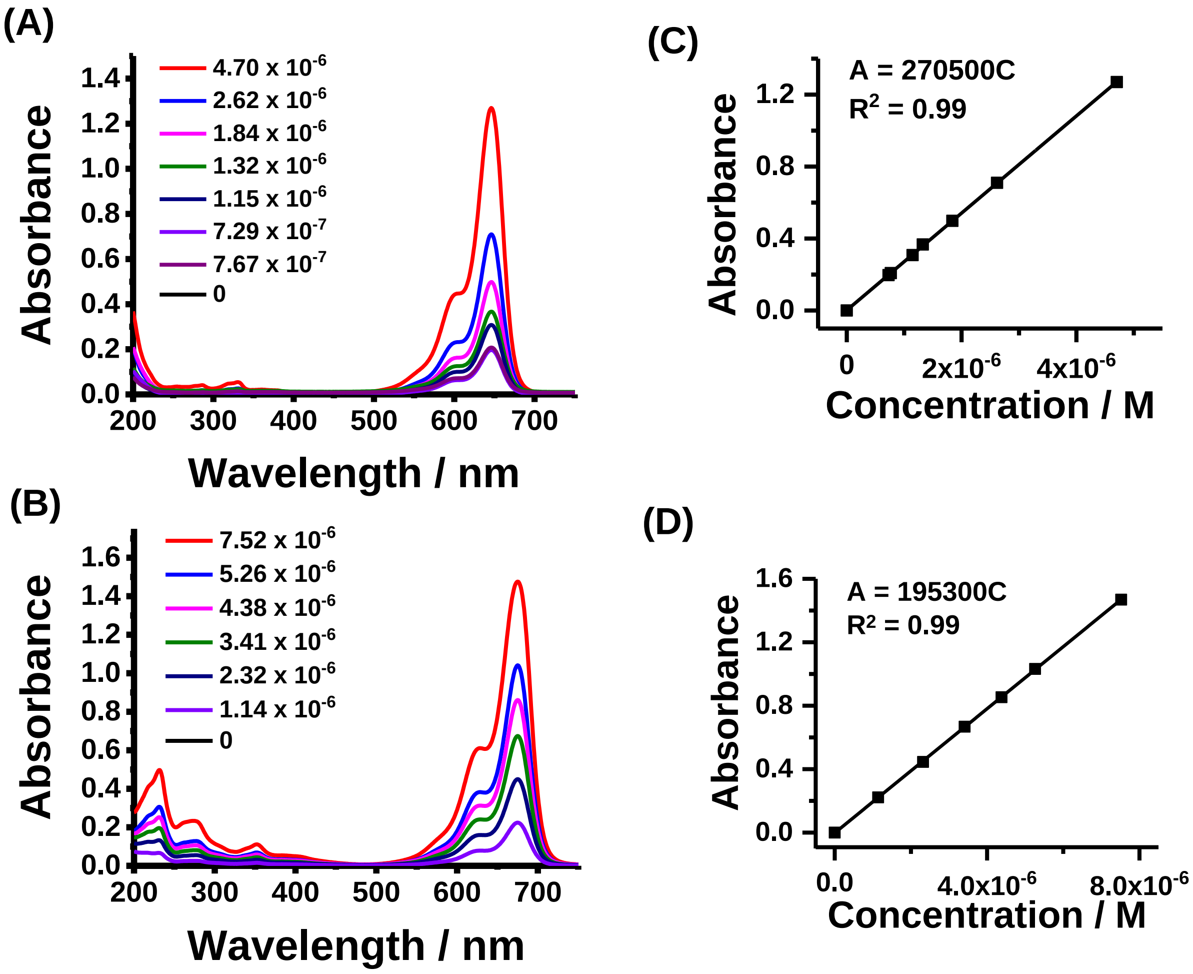

Figure S6. UV-visible spectra for **(A)** Cy5-NH_2 and **(B)** Cy5.5-NH_2 dissolved in water at various concentrations (in M). The calibration data measured for Cy5-NH_2 using absorbance values at 646 nm is shown in **(C)**. The calibration data measured for Cy5.5-NH_2 using absorbance values at 673 nm is shown in **(D)**.

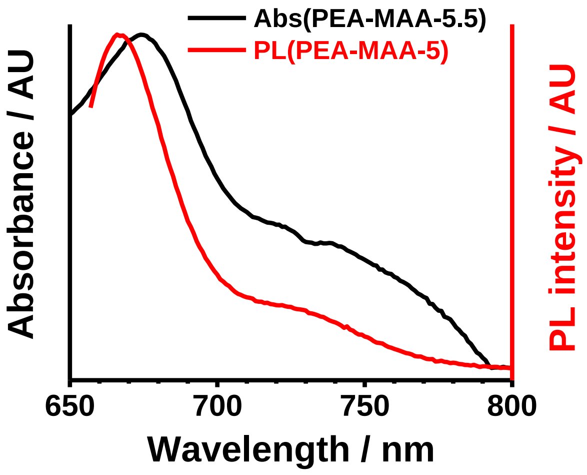

Figure S7. PL spectrum of PEA-MAA-5 and UV-visible spectrum of PEA-MAA-5.5. The strong overlap shows that Cy5 and Cy5.5 can act as a donor and acceptor for NRET within PEA-MAA-5/5.5 particles.

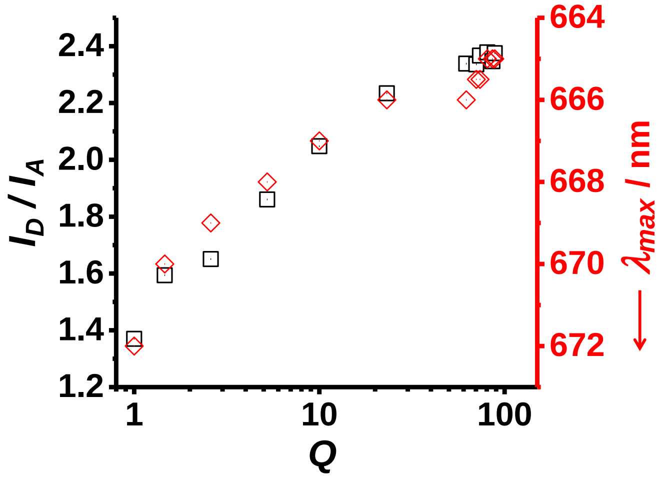

Figure S8. Variation of the PL intensity ratio of the donor and acceptor peaks (I_D/I_A) and λ_{max} with Q for PEA-MAA-5/5.5 MG. Note that the λ_{max} values are plotted in reverse order.

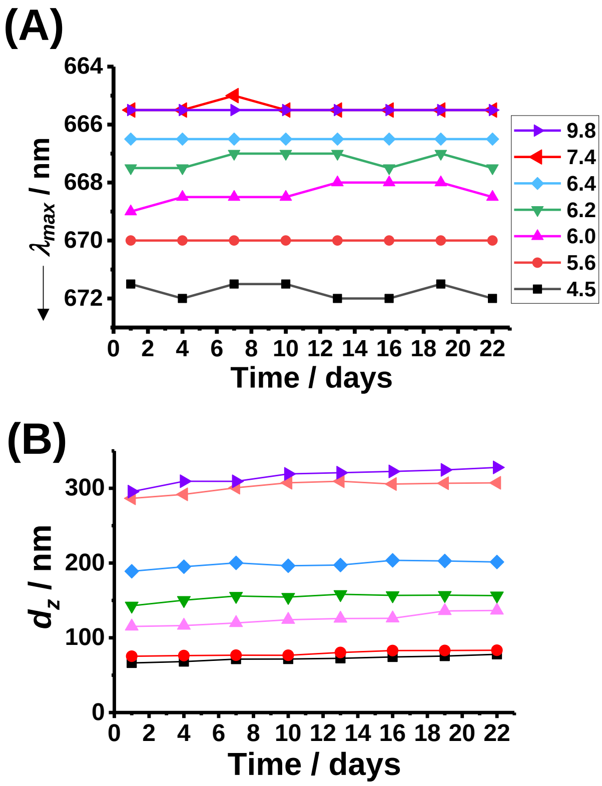

Figure S9. Variation of **(A)** λ_{max} and **(B)** d_z for PEA-MAA-5/5.5 with time measured for a range of pH values (shown). The dispersions were stored at room temperature in the dark between measurements. The legend in (A) also applies to (B).

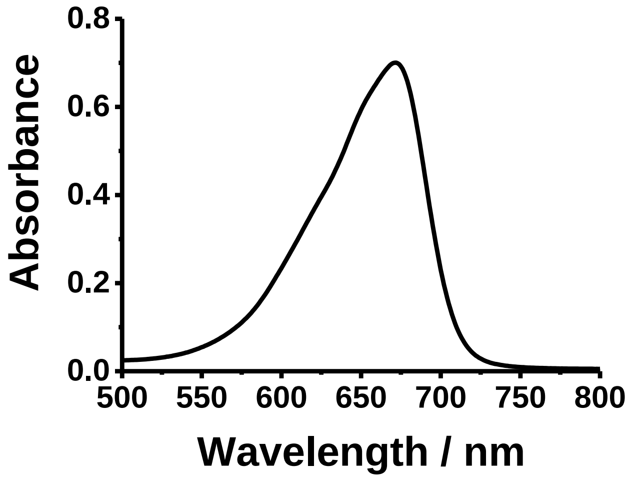

Figure S10. UV-visible spectra for the PNP-5/5.5 dispersion at 25 °C.

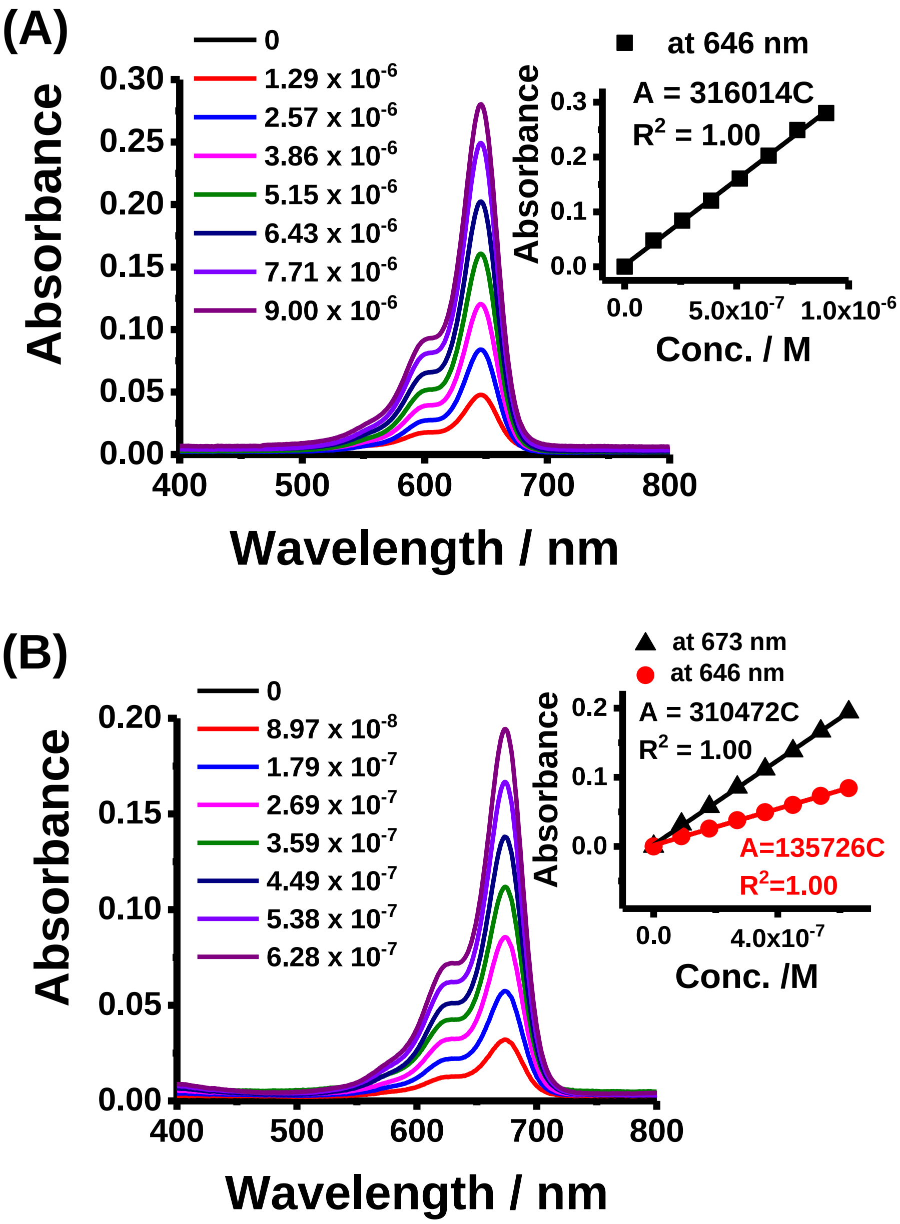

Figure S11. UV-visible spectra and calibration curves used for **(A)** Cy5-NHS and **(B)** Cy5.5-NHS dissolved in water. The concentrations are shown in the legends (M). The insets show the measured absorbance values as a function of fluorophore concentration.

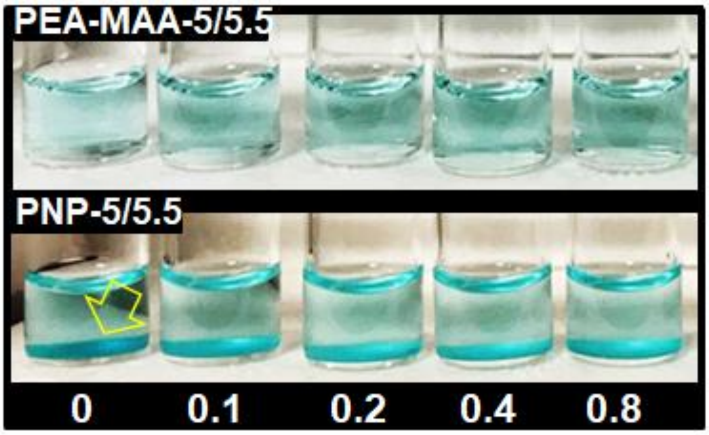

Figure S12. Digital photographs of PEA-MAA-5/5.5 (top row) and PNP-5/5.5 (bottom row) dispersions in the presence and absence of added NaCl. (The concentrations are shown in M.) The dispersions contained pH 7.4 buffer (0.10 M) and the temperature was 37 °C. The yellow arrow highlights sedimented aggregates that are present in all of the tubes containing PNP-5/5.5.

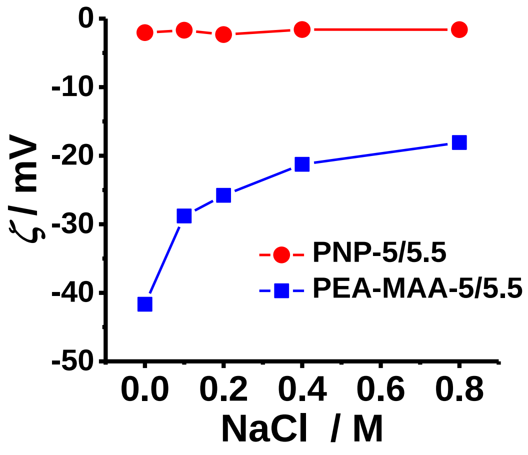

Figure S13. Variation of zeta potential for PEA-MAA/5.5 and PNP-5/5.5 dispersions as a function NaCl concentration. The dispersions contained pH 7.4 buffer (0.10 M) and the temperature was 37 °C.

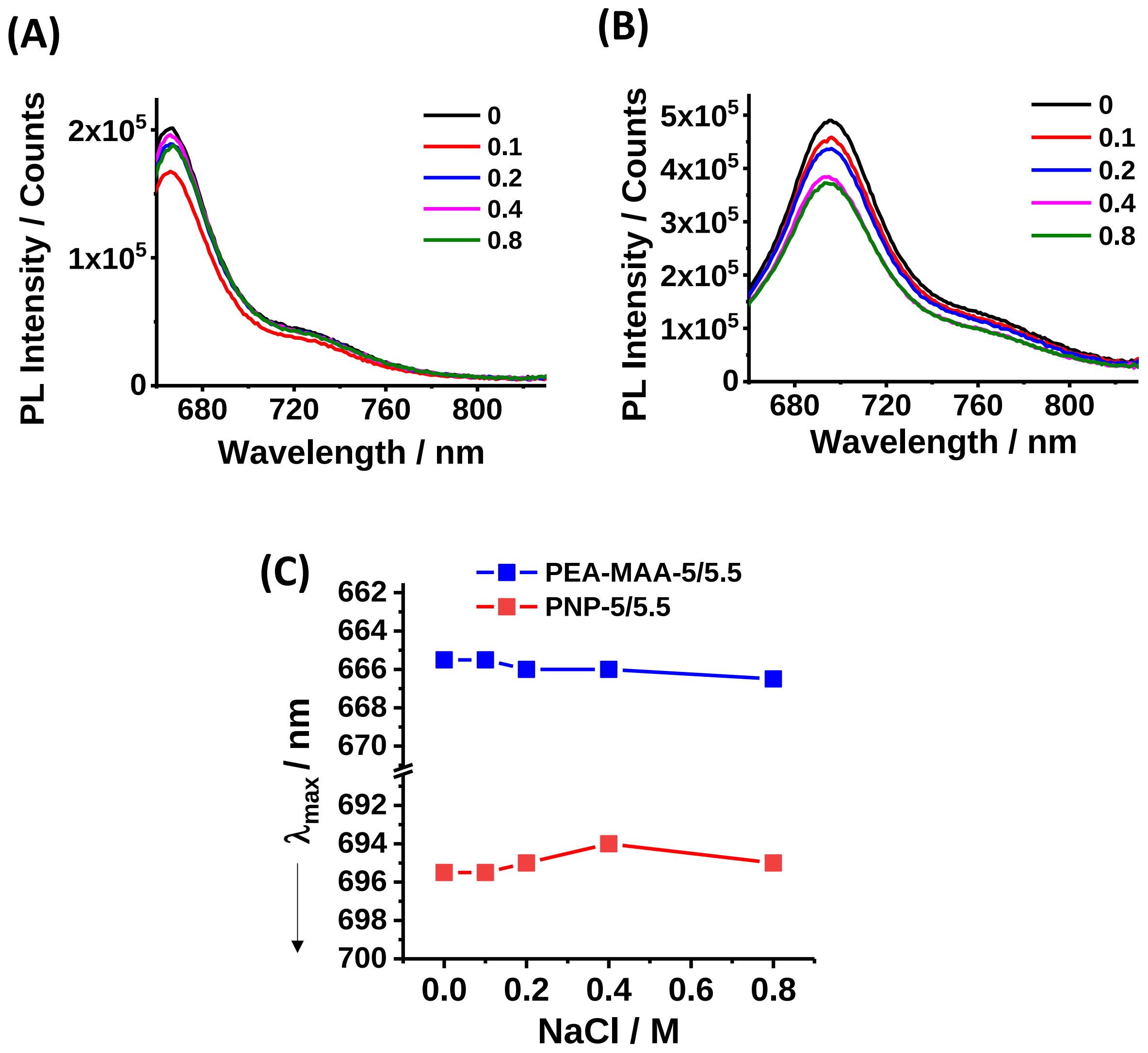

Figure S14. PL spectra for **(A)** PEA-MAA-5/5.5 and **(B)** PNP-5/5.5 obtained using different NaCl concentrations (shown in M) at pH 7.4 and 37 ℃. **(C)** λ_{max} values obtained from (A) and (B).

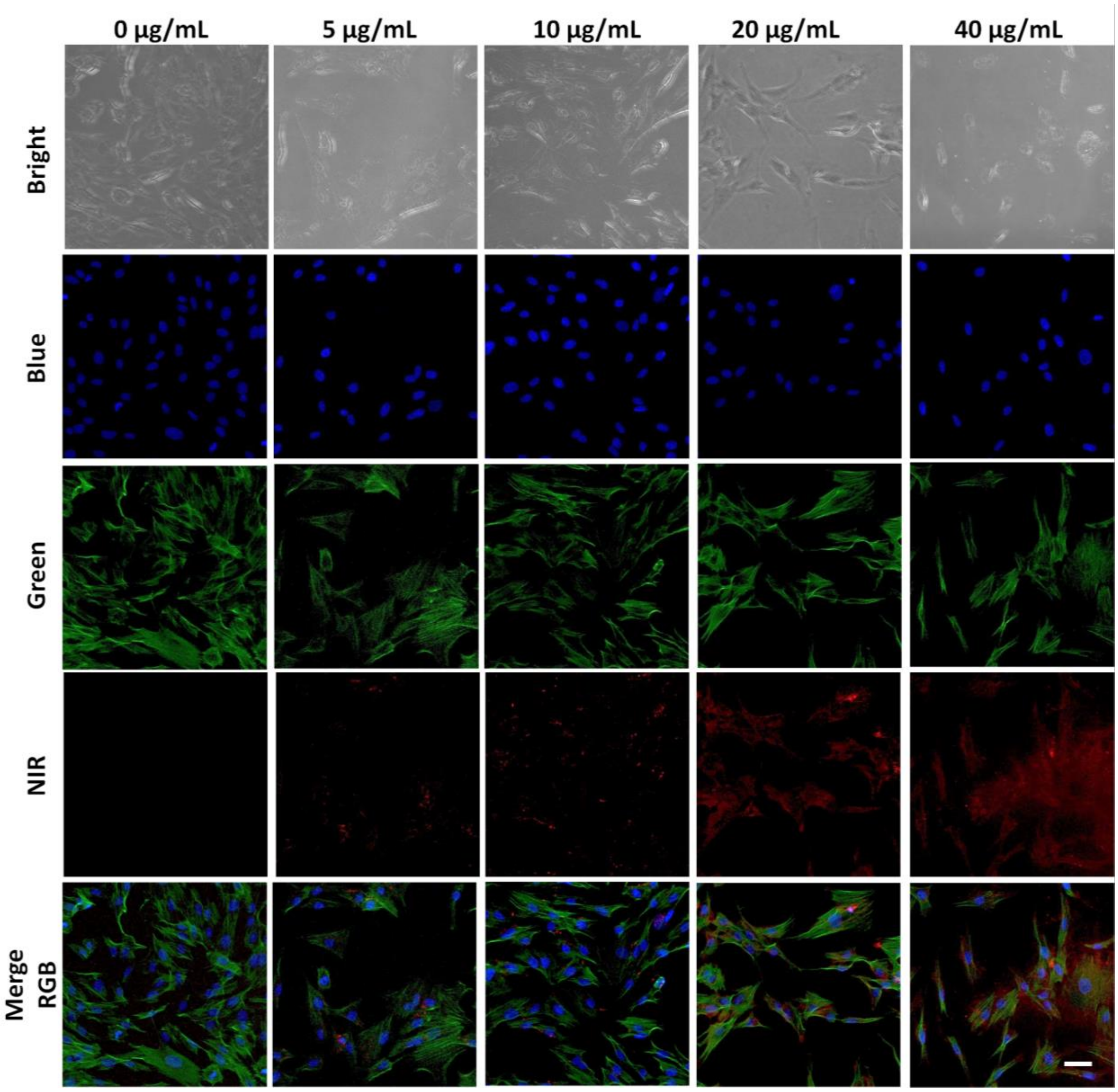

Figure S15. CLSM images of PEA-MAA-5/5.5 particles (0, 5, 10, 20 and 40 μg/mL) following incubation with stem cells. Different and merged (bottom row) colour channels are shown. The medium pH was 6.4. The top row shows the bright field white light images. The scale bar is 50 μm and applies to all images.

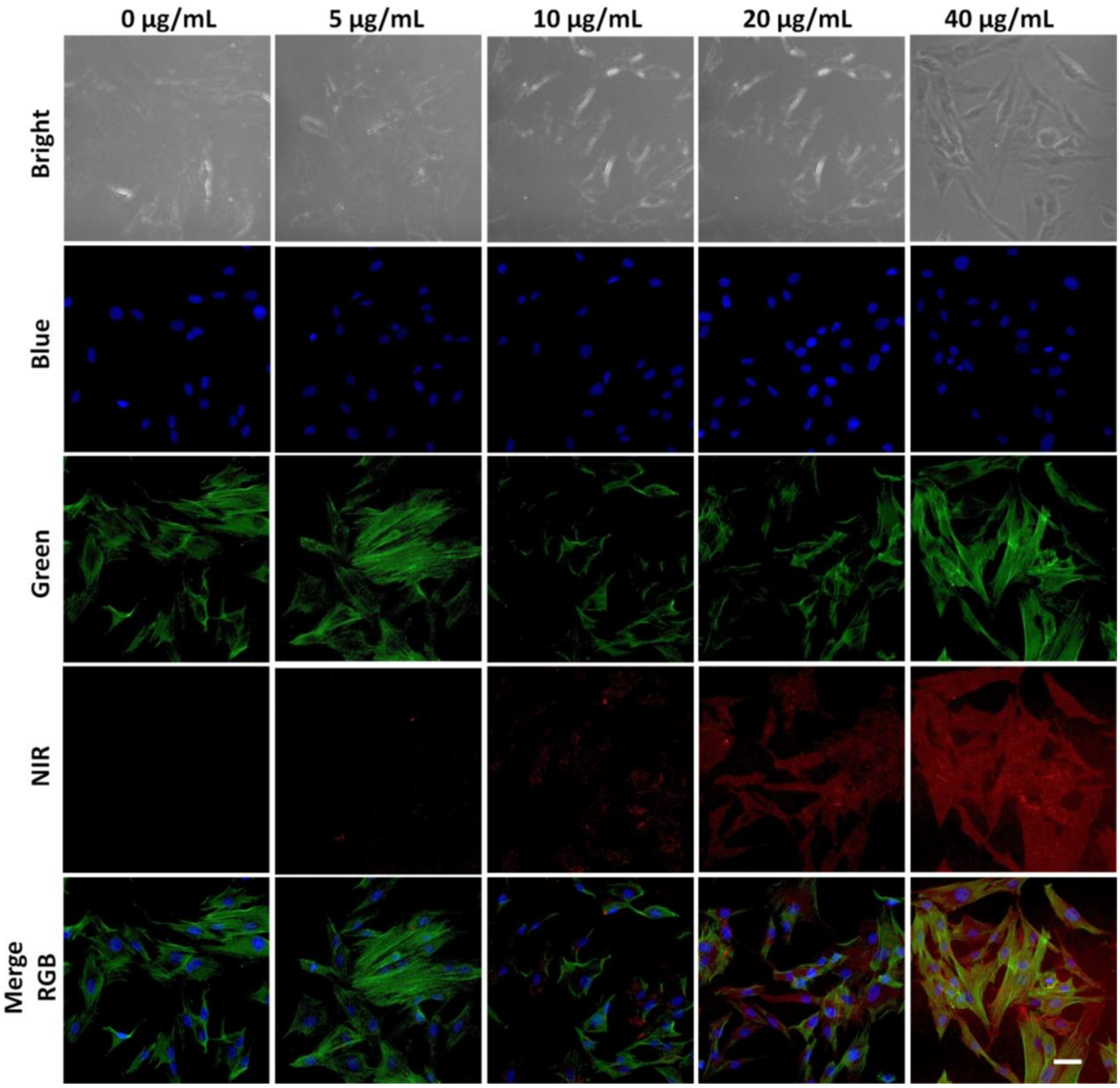

Figure S16. CLSM images of PEA-MAA-5/5.5 particles (0, 5, 10, 20 and 40 μg/mL) following incubation with stem cells. Different and merged (bottom row) colour channels are shown. The medium pH was 7.4. The top row shows the bright field white light images. The scale bar is 50 μm and applies to all images.

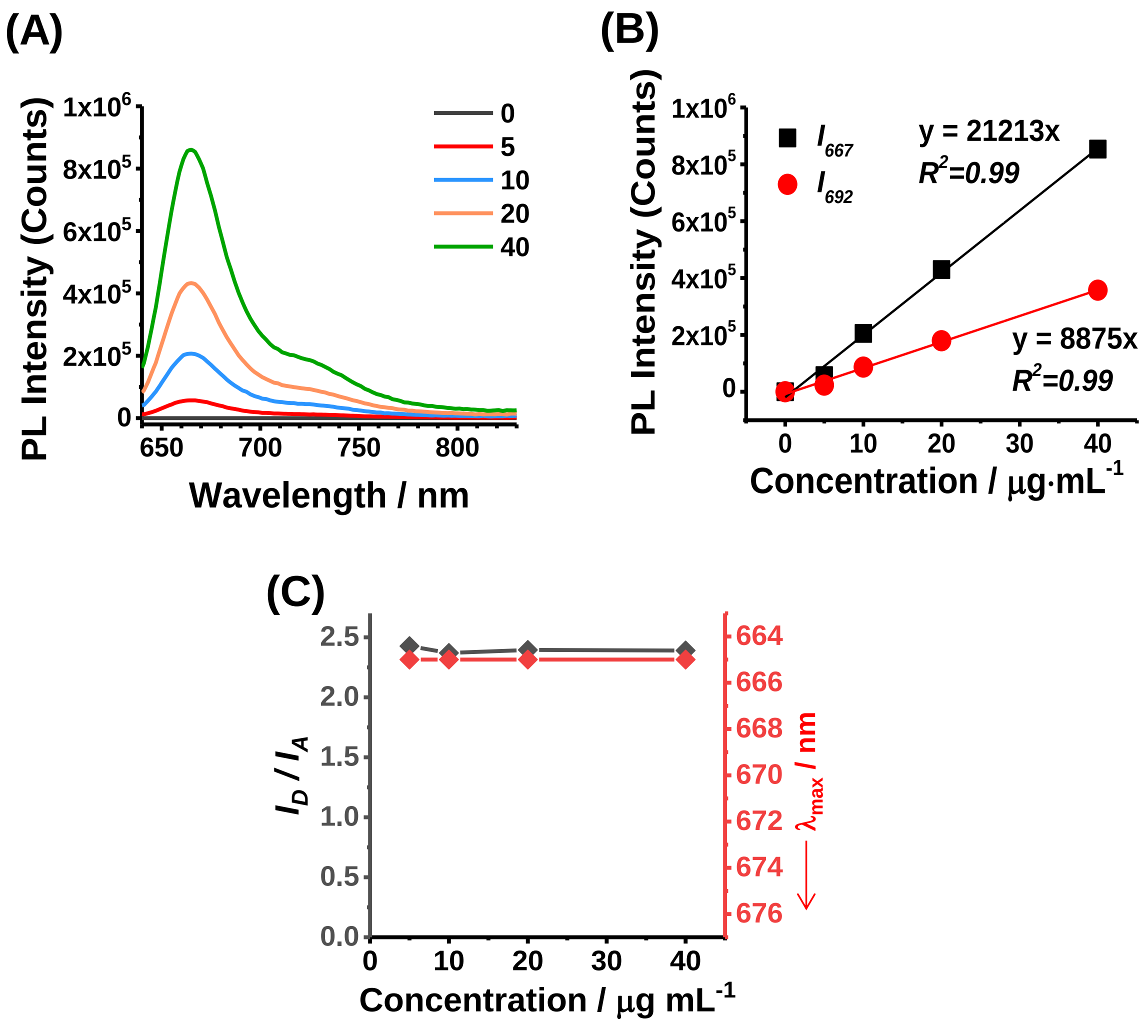

Figure S17. (A) PL spectra for PEA-MAA-5/5.5 dispersions measured using various particle concentrations (im µg/mL)) at pH 7.4. **(B)** PL intensity at 667 and 692 nm versus particle concentration from the spectra in (A). (**C**) I_D/I_A and λ_{max} values from the spectra in (A).

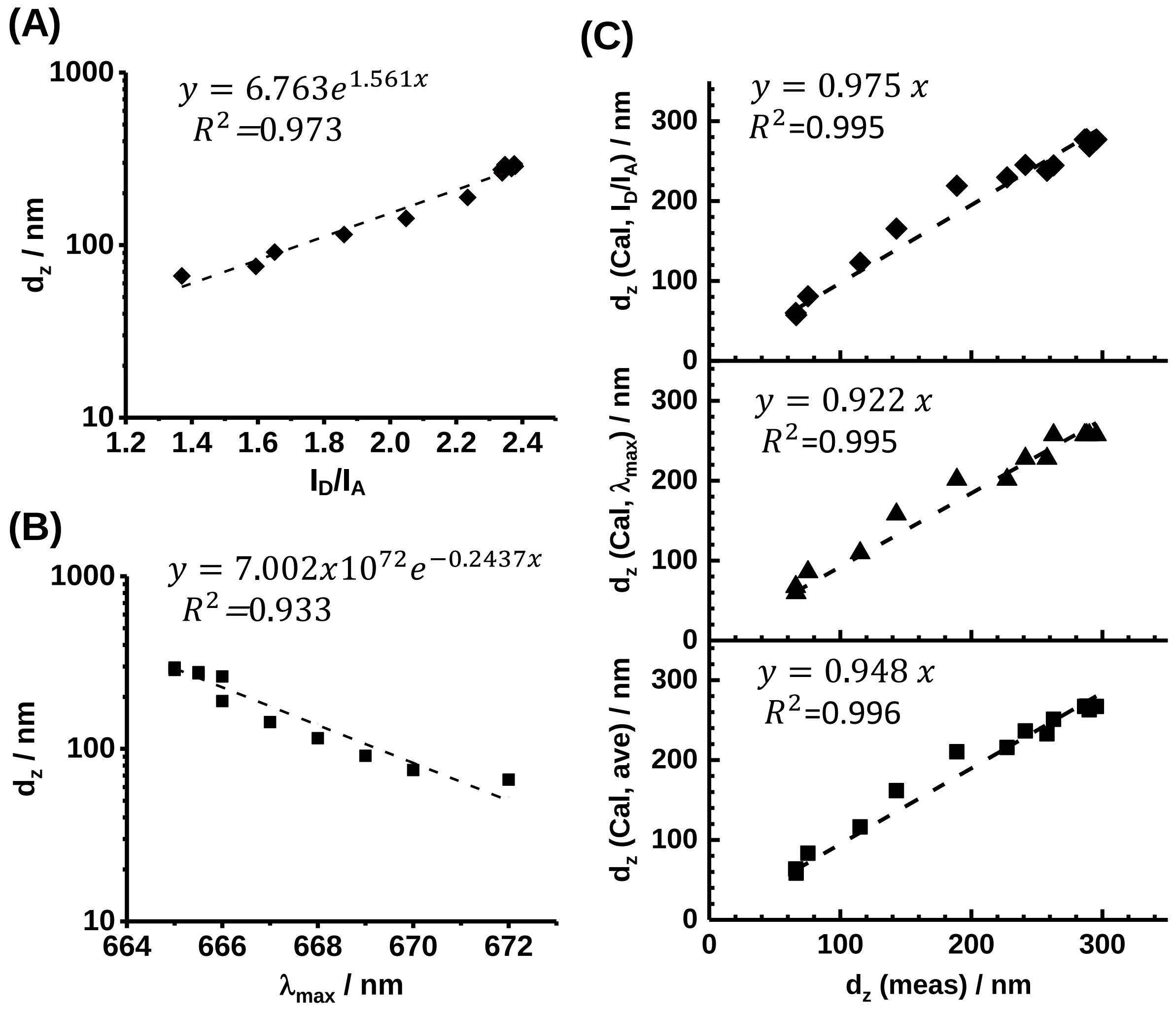

Figure S18. (A) Calibration curve using d_z and I_D/I_A data from figures 1B and 2B. **(B)** Calibration curve using d_z and λ_{max} data from figures 1B and 2B. **(C)** *Calculated* d_z values as a function of *measured* d_z values for the PEA-MAA-5/5.5 data shown in figures 2C (cycle 1 and 2), 2D (1 day), S8 (1 day) and 3E (all concentrations). The calculated values were obtained using the fit in A (top), B (middle) and the average values of the values obtained from A and B (bottom).

TABLES

Table S1. Compositions of the MG particles.

Microgel	MAA / mol% [a]	Cy 5 mol% [b]	Cy 5.5 mol% [b]	d_{TEM} / nm (CV) [c]	$d_{collapse}$ / nm [d]
**PEA-MAA-5/5.5**	41.1	0.010	0.0020	57 (10)	66
**PEA-MAA-5**	41.1	0.010	-	61 (16)	-
**PEA-MAA-5.5**	41.1	-	0.0050	42 (20)	-
**PNP-5/5.5**	-	0.020	0.050	40 (7)	86

[a] Calculated from potentiometric titration data shown in Figure S2A and the fluorophore concentrations. [b] Determined from UV-visible spectroscopy data using the Beer-Lambert law and figures 1C, S4, S5, S9 and S10. [c] Number-average diameters determined from TEM images. The numbers in brackets are the coefficient of variation. [d] z-average diameter measured in the collapsed state for the MGs.

Table S2. Performance comparison for ratiometric photoluminescence stimuli-responsive nanoscale probes based on NRET.

**Symbol** [a]	**Response**[b]	λ_{ex} / **nm**[c]	**Donor/ Acceptor** λ_{em} **nm/nm**[d]	d_h **(collapsed) / nm**[e]	d_h **(swollen) / nm**[f]	Q^g	**Ref**
A	pH/Mg^{2+}	254 or 365	413/513	46	66	3.0	3
B	pH/Ca^{2+}	298	366/414	24	43	5.8	4
C	T	298	370/413	70	160	12	5
D	T	405	470/625	65	225	41	6
E	pH	450	500/620	125	137	1.3	7
F	T	454	610/670	150	350	13	8
G	T/Sugar	470	532/578	43	100	13	9
H	T/Light	470	527/588 or 529/590	30	56	6.5	10
I	T/K^+	470	529/588	46	120	18	11
J	T/Light	470	525/620	75	130	5.2	12
K	T/pH	520	570/670	82	145	5.5	13
L	T	646	671/697	61	116	6.9	2
This work	pH	650	667/692	66	296	90	This work

[a] Symbol used in the legend of Figure 6. [b] pH, T, sugar and light indicate pH-responsive, temperature-responsive, sugar-responsive and light-responsive behaviours for the probes, respectively. [c] Excitation wavelengths. [d] Donor / acceptor emission wavelengths. [e] Hydrodynamic diameter in the collapsed state from DLS measurements. [f] Hydrodynamic diameter in the swollen state from DLS. [g] Particle swelling ratio calculated from the volume of the swollen and collapsed particles using the values in the fifth and sixth columns.

REFERENCES

1. S. Fazal, B. Paul-Prasanth, S. V. Nair and D. Menon, *Acs Appl Mater Inter*, 2017, **9**, 28260-28272.
2. C. D. Jones, J. G. McGrath and L. A. Lyon, *J Phys Chem B*, 2004, **108**, 12652-12657.
3. M. N. Zhu, D. D. Lu, S. L. Wu, Q. Lian, W. K. Wang, L. A. Lyon, W. G. Wang, P. Bartolo and B. R. Saunders, *Nanoscale*, 2019, **11**, 11484-11495.
4. M. N. Zhu, D. D. Lu, S. L. Wu, Q. Lian, W. K. Wang, A. H. Milani, Z. X. Cui, N. T. Nguyen, M. Chen, L. A. Lyon, D. J. Adlam, A. J. Freemont, J. A. Hoyland and B. R. Saunders, *Acs Macro Lett*, 2017, **6**, 1245-1250.
5. D. J. Gan and L. A. Lyon, *J Am Chem Soc*, 2001, **123**, 8203-8209.
6. J. Liu, X. D. Guo, R. Hu, J. Xu, S. Q. Wang, S. Y. Li, Y. Li and G. Q. Yang, *Anal Chem*, 2015, **87**, 3694-3698.
7. H. S. Peng, J. A. Stolwijk, L. N. Sun, J. Wegener and O. S. Wolfbeis, *Angew Chem Int Edit*, 2010, **49**, 4246-4249.
8. F. Pinaud, R. Millereux, P. Vialar-Trarieux, B. Catargi, S. Pinet, I. Gosse, N. Sojic and V. Ravaine, *J Phys Chem B*, 2015, **119**, 12954-12961.
9. D. Wang, T. Liu, J. Yin and S. Y. Liu, *Macromolecules*, 2011, **44**, 2282-2290.
10. J. Yin, H. B. Hu, Y. H. Wu and S. Y. Liu, *Pol. Chem.*, 2011, **2**, 363-371.
11. J. Yin, C. H. Li, D. Wang and S. Y. Liu, *J Phys Chem B*, 2010, **114**, 12213-12220.
12. S. H. Lee, H. T. Bui, T. P. Vales, S. Cho and H. J. Kim, *Dyes Pigments*, 2017, **145**, 216-221.
13. Y. L. Chiu, S. A. Chen, J. H. Chen, K. J. Chen, H. L. Chen and H. W. Sung, *Acs Nano*, 2010, **4**, 7467-7474.
